# Transcriptional signature of islet neogenesis-associated protein peptide-treated rat pancreatic islets reveals induction of novel long non-coding RNAs

**DOI:** 10.3389/fendo.2023.1226615

**Published:** 2023-09-29

**Authors:** Agustín Romero, Ana C. Heidenreich, Carolina L. Román, Macarena Algañarás, Ezequiel Nazer, Juan J. Gagliardino, Bárbara Maiztegui, Luis E. Flores, Santiago A. Rodríguez-Seguí

**Affiliations:** ^1^ Instituto de Fisiología, Biología Molecular y Neurociencias (IFIBYNE), CONICET-Universidad de Buenos Aires, Ciudad Universitaria, Buenos Aires, Argentina; ^2^ Departamento de Fisiología, Biología Molecular y Celular, Facultad de Ciencias Exactas y Naturales, Universidad de Buenos Aires, Buenos Aires, Argentina; ^3^ Centro de Endocrinología Experimental y Aplicada (CENEXA) - Universidad Nacional de La Plata (UNLP) - CONICET- Centro Asociado a la Comisión de Investigaciones Científicas de la Provincia de Buenos Aires (CeAs CICPBA), Facultad de Ciencias Médicas UNLP, La Plata, Argentina

**Keywords:** INGAP, islet, rat, pancreas, beta cell (β-cell), regeneration, long noncoding RNA (IncRNA), *Ri-lnc1*

## Abstract

**Background:**

Diabetes mellitus is characterized by chronic hyperglycemia with loss of β-cell function and mass. An attractive therapeutic approach to treat patients with diabetes in a non-invasive way is to harness the innate regenerative potential of the pancreas. The Islet Neogenesis-Associated Protein pentadecapeptide (INGAP-PP) has been shown to induce β-cell regeneration and improve their function in rodents. To investigate its possible mechanism of action, we report here the global transcriptional effects induced by the short-term INGAP-PP *in vitro* treatment of adult rat pancreatic islets.

**Methods and findings:**

Rat pancreatic islets were cultured *in vitro* in the presence of INGAP-PP for 4 days, and RNA-seq was generated from triplicate treated and control islet samples. We performed a *de novo* rat gene annotation based on the alignment of RNA-seq reads. The list of INGAP-PP-regulated genes was integrated with epigenomic data. Using the new gene annotation generated in this work, we quantified RNA-seq data profiled in INS-1 cells treated with IL1β, IL1β+Calcipotriol (a vitamin D agonist) or vehicle, and single-cell RNA-seq data profiled in rat pancreatic islets. We found 1,669 differentially expressed genes by INGAP-PP treatment, including dozens of previously unannotated rat transcripts. Genes differentially expressed by the INGAP-PP treatment included a subset of upregulated transcripts that are associated with vitamin D receptor activation. Supported by epigenomic and single-cell RNA-seq data, we identified 9 previously unannotated long noncoding RNAs (lncRNAs) upregulated by INGAP-PP, some of which are also differentially regulated by IL1β and vitamin D in β-cells. These include *Ri-lnc1*, which is enriched in mature β-cells.

**Conclusions:**

Our results reveal the transcriptional program that could explain the enhancement of INGAP-PP-mediated physiological effects on β-cell mass and function. We identified novel lncRNAs that are induced by INGAP-PP in rat islets, some of which are selectively expressed in pancreatic β-cells and downregulated by IL1β treatment of INS-1 cells. Our results suggest a relevant function for *Ri-lnc1* in β-cells. These findings are expected to provide the basis for a deeper understanding of islet translational results from rodents to humans, with the ultimate goal of designing new therapies for people with diabetes.

## 1 Introduction

Diabetes is a metabolic disorder characterized by chronic hyperglycemia that arises from multiple pathophysiological causes (1), its most frequent forms being type 1 and type 2 diabetes (T1D and T2D). While T1D occurs as a consequence of a massive loss of insulin-producing β-cells caused by an autoimmune attack, T2D is due to a combination of insulin resistance and inadequate insulin secretion resulting from a decreased β-cell mass and/or function. As none of the available therapeutics can positively modulate β-cell mass, the solution for people with T1D to replenish their lost β-cells is still the transplantation of pancreatic islets from cadaveric donors (2). However, the high demand and scarcity of this tissue have led to the search for alternative approaches to recover β-cell mass and function (3). One strategy involves the generation and transplantation of new β-cells derived *in vitro* from human pluripotent stem cells (4). Although researchers are making progress towards producing fully functional β-cells, current protocols still require improvement, primarily to ensure the functionality of these cells when transplanted into humans, and to prevent their rejection by the host’s immune system. An appealing and less invasive therapeutic approach aims to harness the innate regenerative potential of the pancreas (5). Identifying molecules capable of stimulating β-cell regeneration, along with a better understanding of the pancreatic cell types involved in this process, becomes crucial for achieving an effective regenerative therapy in humans (6).

The Islet Neogenesis Associated Protein (INGAP) has been demonstrated to induce β-cell regeneration in hamster, rat, and mouse animal models (7–10). The primary biological effects are also achieved with a pentadecapeptide with the amino acid sequence 104-118 of the INGAP molecule (INGAP-PP) (8, 11).

Previous reports have shown that treating rat pancreatic islets with INGAP-PP *in vitro* induces the expression of genes directly related to the secretory function of β-cells (11–15). This treatment enhances insulin secretion in response to glucose, amino acids, KCl, and tolbutamide and increases calcium intracellular concentrations (15, 16), which may be due to the activation of the PI3-K/AKT pathway (17). INGAP-PP treatment also leads to increased β-cell replication and neogenesis while decreasing its rate of apoptosis (11). Recent findings have also highlighted that INGAP-PP exerts an anti-inflammatory effect on β-cells (18) and promotes islet angiogenesis (19, 20).

Notably, transgenic mice with overexpression of INGAP are resistant to the diabetogenic action of streptozotocin (STZ), which improves oral glucose tolerance and delays the onset of diabetes (9, 21). Additionally, administering INGAP-PP to mice with STZ-induced diabetes increased their β-cell mass and reduced glycemia in 50% of cases (8). Although INGAP-PP treatment in humans did not yield definitive conclusions, it did show improvement in some parameters assessed, such as arginine-stimulated C-peptide secretion in the T1D trial and HbA1C levels in the T2D trial (22).

Current efforts focus on enhancing the biological effects of INGAP-PP by developing better-tolerated formulations to achieve an optimal clinical response (23). New patents have also been filed, describing modified INGAP- and Reg3-based sequences (24–28). Moreover, clinical trials involving INGAP-PP treatment in diabetic patients have been conducted since 2009, with the most recent one completed in March 2017 (source: Clinicaltrials.gov, Identifier NCT02204397). Thus, INGAP-PP treatment for improving the condition of T1D patients is still a field of intense research that is actively being translated to the industry and community through intellectual property protection and clinical trials.

Despite considerable effort devoted to elucidating the pathways modulated by INGAP-PP treatment over the last decades, its mechanism of action remains largely elusive to date. Importantly, INGAP-PP/Reg3 peptides have been shown to elicit cell responses in a diverse group of cell types, including pancreatic β-cells, ductal cells, and endothelial cells (19, 20, 29–31). As such, the desired ultimate effect of enhanced insulin secretion and β-cell regeneration might arise from a complex intercellular signaling that is coordinately induced by INGAP-PP treatment across the different cell types present in the islet microenvironment. To shed light on this matter, we present here the global transcriptional effects induced by short-term *in vitro* INGAP-PP treatment of adult rat pancreatic islets.

Based on a *de novo* gene annotation we identified 1,669 differentially expressed genes after INGAP-PP treatment, including dozens of unannotated rat transcripts. Integration with epigenomic data suggests that INGAP-PP might coordinate the activation of Hif1a-, Nfat- and Vitamin D receptor-regulated programs. Indeed, INGAP-PP upregulates a subset of genes associated with the β-cell protective effects of vitamin D against interleukin 1β (IL1β)-induced stress. Our analyses also bolstered the identification of previously non-annotated long non-coding RNAs (lncRNAs) that are upregulated by INGAP-PP, some of which are not conserved in mice or humans. These include *Ri-lnc1*, which is enriched in β-cells and could be potentially used as a novel mature β-cell marker. Taken together, the results presented here reveal novel genes and potential mechanisms that could underlie the positive physiological effects of INGAP-PP on β-cell function and mass.

## 2 Materials and methods

### 2.1 Animals, islet isolation and culture

Adult male Wistar rats were maintained under controlled conditions of 23°C and a fixed 12-hour light, 12-hour dark cycle, with free access to a standard commercial diet and water. Experiments were performed according to the “Ethical principles and guidelines for experimental animals” (3rd ed., 2005) from the Swiss Academy of Medical Sciences. The animal study was reviewed and approved by Animal Welfare Committee (CICUAL) of La Plata School of Medicine, UNLP (T01-04-2021). At the time of euthanasia by cervical dislocation, the whole pancreas from each animal was removed to isolate islets by collagenase digestion (17). Freshly isolated islets were cultured in RPMI-1640 media for 4 days as described (17), in the absence (C, control) or presence of 50 μg/mL INGAP-PP (I). Insulin secretion after treatment was determined by radioimmunoassay as described (17). The INGAP-PP (NH-Ile-Gly-Leu-His-Asp-Pro-Ser-His-Gly-Thr-Leu-Pro-Asn-Gly-Ser-COOH) was kindly provided by Dr. G.A. Fleming (Kinexum LLC, West Virginia) or purchased from GL Biochem (Shanghai, China). Quality control of the peptide (amino acid analysis and mass spectrometry) indicated greater than 95% purity and a molecular weight of 1501.63.

### 2.2 RNA-seq

Total RNA was isolated using the RNeasy Mini Kit (Qiagen, #74106). DNA contamination was avoided by treating samples with DNase I (Invitrogen). RNA from triplicate INGAP-PP and untreated samples was assessed for quality (RNA integrity number > 9) by Agilent bioanalyzer. Paired-end RNA-seq libraries were sequenced on an Illumina HiSeq4000, obtaining >92 million 100bp reads. Read alignment and TPM gene expression quantification were performed with the HISAT-StringTie analysis pipeline (32), which allowed for *de novo* annotation of genes expressed in rat tissues. For this, we used public sample triplicates for rat brain, liver and pancreatic islets, as well as our triplicate RNA-seq samples sequenced for INGAP-PP treated and untreated rat pancreatic islets (**Supplementary Table 1**). Additional details are provided in **Supplementary Methods**. Downstream analyses included Clustering and Principal Component Analysis (PCA), which were performed following previously described methods (33). The differential expression of genes was determined by modeling the INGAP-PP effect with a linear mixed effect (LME) model to account for the random effects of different batches of rat donors as previously described (34). Gene Ontology (GO) and Gene Set Enrichment Analysis (GSEA) were performed as described (33). Further information, including the pipeline followed for characterization of rat unannotated genes and homolog gene search, is provided in **Supplementary Methods**.

### 2.3 Single-cell RNA-seq

Single-cell RNA-seq data from dissociated mouse pancreatic islets was taken from Baron et al. (35) Already quantified and cell-type clustered data was downloaded from the Gene Expression Omnibus (GEO) with accession GSE84133. Data was processed for visualization of violin plots to show the expression of selected genes using the ScanPy package (36). Single-cell RNA-seq data from dissociated rat pancreatic islets was taken from Vivoli et al. (37) Raw data was downloaded from the Gene Expression Omnibus (GEO) with accession GSE193857. We next built a custom genome reference, based on the “curated rat reference genome annotation” created in this work, using the CellRanger (v.7.1.0) *mkref* function following the steps described in the 10xgenomics webpage for this purpose. Reads from .*fastq* files were aligned using the Cellranger *count* function using the “curated rat reference genome annotation” as transcriptome reference input file. Data from triplicate vehicle and palmitate samples, and quadruplicate oleate samples, were re-quantified with Cellranger as described above. Downstream analyses were performed with Seurat (v.4.0.4). These included single cell filtering (nFeature between 2000 and 5000, nCount between 5000 and 50000 and mitochondrial percentage lower than 10). Additionally, integration parameters were set to 30 for npcs, 3000 highly variable features were selected and n-neighbors were set to 20. For cluster detection and filtering of non-endocrine clusters, the resolution parameter was set to 0.4. We next selected the β-cells subset for in-depth analysis, excluding a small β-cell subcluster expressing high levels of *Gcg* and *Ins2*. The remaining β-cells were reanalyzed using 3000 highly variable features for data integration, npcs=30, and the Seurat *FindNeighbors* and *FindClusters* functions with default parameters. The resulting clusters were next merged according to their average expression of *Ri-lnc1* to obtain 3 new clusters containing cells with high, medium or low *Ri-lnc1* expression levels. To define such clusters the upper threshold value was set at 0.3 and the lower threshold value was set at 0.18.

### 2.4 ChIP-seq and ATAC-seq

Publicly available ChIP-seq and ATAC-seq raw datasets were obtained from the Sequence Read Archive (SRA) database (**Supplementary Table 1**). Sequence reads were realigned to the rat (rn6) genome and further processed as previously described (38, 39), with detailed information provided in **Supplementary Methods**. The definition for identification of promoter and enhancer regions in INS-1 is described in detail in **Supplementary Methods**. The heatmap and aggregation (averageogram) plots were computed as described (33, 38).

### 2.5 Motif discovery

*De novo* motif discovery using a window of 500 bp centered at the INGAP-PP enhancer effectors (defined by the ATAC-seq peak coordinates) was performed as described (38, 39). **Supplementary Methods** include detailed information for the differential motif enrichment analysis within the regions of interest, using different sets of background regions.

### 2.6 Statistical analysis

Statistical significance was assessed using the Wilcoxon rank-sum test, EdgeR, Student’s t test or a linear mixed effect model as indicated in each case, with *p<*0.05 considered significant. Hypergeometric tests were conducted utilizing the total number of genes from our curated genome annotation (see **Supplementary Methods**). All results are given as means ± SEM. All statistical analysis and visualization were done with R and Bioconductor package.

## 3 Results

### 3.1 Global transcriptional response of rat pancreatic islets treated with INGAP-PP based on a *de novo* transcript annotation

**Figure 1:**
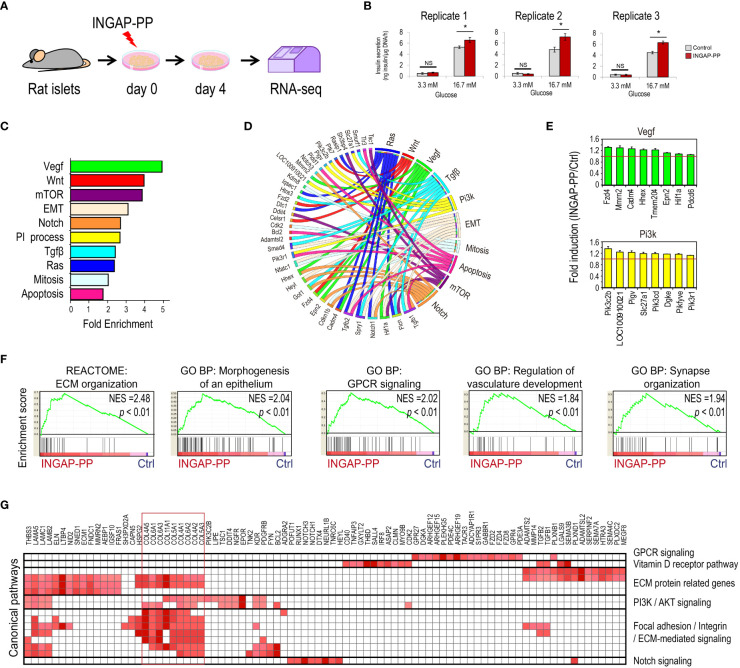
Analysis of the pancreatic islet transcriptional response to the INGAP-PP treatment. **(A)** Experimental design. **(B)** Insulin secretion in response to 3.3 and 16.7 mM glucose after *in vitro* INGAP-PP treatment of rat pancreatic islets. Insulin released into the incubation media was measured by RIA in biological triplicates and it was expressed as ng of insulin per µgDNA/1 h. Bars represent means ± SEM from three independent measurements made for each treatment replicate using medium samples obtained from different islet aliquots. *p<0.05; NS, Not significant. **(C)** Functional annotation of the INGAP-PP-regulated genes. *p<*0.05 for all categories shown. **(D)** Circus plot showing the association of genes with its corresponding functional annotations. **(E)** Average gene expression fold enrichment for top enriched genes in selected annotations shown in **(C)** Bars represent means ± SEM. **(F)** Selected Gene Set Enrichment Analysis (GSEA) results. All results presented are significant, considering a *p<*0.05 and FDR< 0.25. **(G)** Heatmap depicting the Leading Edge Analysis of the top enriched GSEA categories related to the expression of ECM proteins reveals a large overlap in the genes associated with these categories.

To study the molecular programs underlying the enhancement of β-cell mass and function following INGAP-PP treatment, isolated rat pancreatic islets were cultured in the presence of this peptide for 4 days (**Figure 1A**). This protocol has been extensively used in our group to evaluate the INGAP-PP effects on hamster and rat pancreatic islets (16, 17, 19, 40, 41). We generated three INGAP-PP-treated and control islet samples following this approach, and the peptide effect was validated by quantifying glucose-stimulated insulin secretion (GSIS) by radioimmunoassay (RIA) (**Figure 1B**). We next performed bulk RNA-seq on these islet samples with validated INGAP-PP outcomes.

Compared to the mouse and human genomes, the rat genome is still poorly annotated. Thus, we performed a *de novo* rat gene annotation based on the alignment of RNA-seq reads using the HISAT-StringTie pipeline (32), which was then used to quantify gene expression in each sample and replicate. This approach allowed the detection of 14,701 genes expressed in islets (>0.5 TPM in either control or INGAP-PP samples), including 1,266 unannotated rat transcripts (**Supplementary Table 2**). Unannotated genes were labeled as MSTRG followed by a unique number identifying each transcript. This initial, more accurate rat islet transcriptomic quantification was used to explore the INGAP-PP treatment effects.

A global transcriptomic analysis discriminated samples by tissue (**Supplementary Figures 1A-C**), but also revealed that islet samples were first clustered by replicate, rather than treatment. This observation suggests that replicate transcriptomic variability in untreated islet samples dominates over the transcriptional effects induced by INGAP-PP. Previous reports have shown that donor variability can hinder islet treatment or phenotype effects due to the heterogeneous cellular composition of pancreatic islets (34, 35, 42). Indeed, further exploration of our replicate samples for cell type-specific markers, taken from well-characterized single-cell RNA-seq (scRNA-seq) profiles (35), confirmed an important variability in the expression level of endothelial- (higher in replicate 3 samples) and macrophage/Schwann/stellate cell-specific markers (overrepresented in samples from replicates 1 and 2, **Supplementary Figure 1D**). Thus, a variable cell type composition in the islets isolated from biological replicates might contribute differently to the RNA-seq signal.

Since INGAP-PP has been reported to signal to several cell types, including endothelial and β-cells (19, 20, 29, 31), a potential variable cell-type composition between replicates required us to consider treatment effects related to their matched control samples. To evaluate the INGAP-PP treatment effects we used a linear mixed-effect model, accounting for the transcriptome variability of the different rat donors as recently reported (34). This approach allowed us to identify transcripts modulated by the peptide treatment (*p*<0.05). After filtering low-expressed genes (<0.5 TPM) we identified a set of 1,669 genes with expression consistently regulated in response to INGAP-PP treatment, most of which (98%, 1,631 out of 1,669) were upregulated. Of these, 75 were previously unannotated rat islet transcripts (**Supplementary Table 3**).

As expected, INGAP-PP-regulated genes were enriched in functional annotations associated with signaling pathways previously shown to be regulated by this peptide, including PI3K (17), VEGF (19), apoptosis (11) and mitosis (29) (**Figures 1C-E**, **Supplementary Table 4**). This analysis also revealed the enrichment of annotations associated with pathways known to play important roles in β-cell function and development, including mTOR, Notch, Wnt and TGF-β (43, 44), suggesting that they are modulated by INGAP-PP. A gene set enrichment analysis (GSEA) supported these results and extended the list of potentially regulated pathways to include the enrichment of ERK, Rho, GPCR and vitamin D receptor signaling (**Figures 1F, G**, **Supplementary Table 5**). Genes associated with blood vessel morphogenesis and related categories were also induced by INGAP-PP treatment (**Supplementary Figures 2A-D**), consistently with a reported role for this peptide in islet angiogenesis (19, 20). Interestingly, the functional annotation of INGAP-PP-induced genes using GSEA was largely dominated by the enrichment of categories related to extracellular matrix (ECM) protein secretion and remodeling, driven by the increased expression of genes coding for collagens (**Figures 1F, G**). Noteworthy, the top enriched genes in these related categories are selectively expressed in activated pancreatic stellate cells (**Supplementary Figure 2E**) (35). Inspection of the genomic loci for these genes, which are not bound by any of the five endocrine-specific transcription factors profiled in human pancreatic islets, further supports INGAP-PP regulation in non-endocrine cells (**Supplementary Figure 2F**). Taken together, these findings suggest that INGAP-PP treatment induces, either directly or indirectly, a transcriptional response in minor islet cell populations which include endothelial and stellate cells.

### 3.2 Genomic programs underlying the effects of INGAP-PP in β-cells

**Figure 2:**
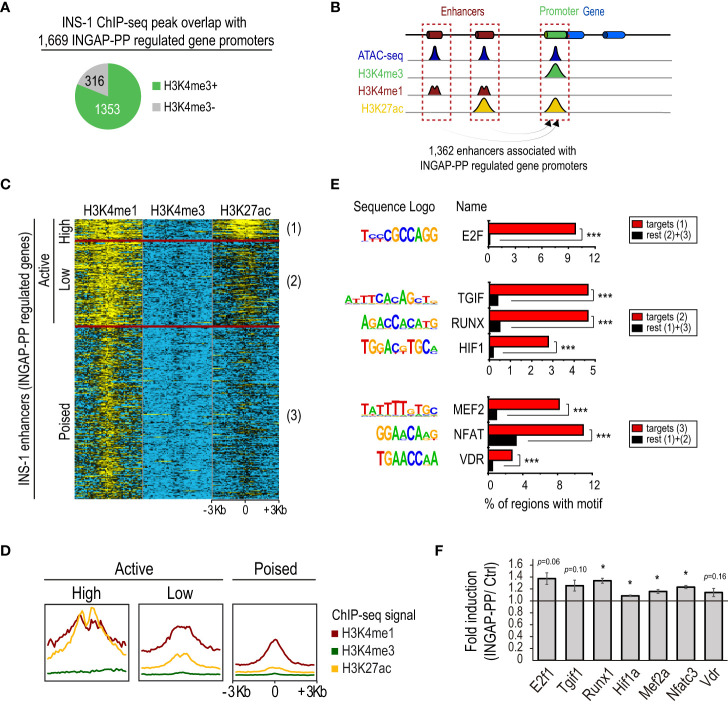
Integrated analysis of the INGAP-PP-regulated genes with epigenomic data profiled in INS-1. **(A)** INS-1 H3K4me3 ChIP-seq peak overlaps with promoters of the 1,669 INGAP-PP-regulated genes. **(B)** Schematic depicting enhancer association to their closest gene promoters. **(C)** Clustered heatmap showing normalized H3K4me3, H3K4me1 and H3K27ac ChIP-seq signals in untreated INS-1 cells within a 6Kb window centered at the ATAC-seq summits of the potential INGAP-PP enhancer effectors. **(D)** Aggregation plots showing H3K4me3, H3K4me1 and H3K27ac ChIP-seq signal enrichments centered (+/- 3Kb) at the INS-1 ATAC-seq summits for active (high), active (low) and poised INS-1 enhancers associated to the promoters of INGAP-PP-regulated genes. **(E)** Selected top *de novo*-motifs recovered from active (high), active (low) and poised INS-1 enhancers shown in **(C)**. **(F)** Average gene expression fold enrichment in rat pancreatic islets for genes coding for the transcription factors shown in **(E)**. Bars represent means ± SEM. Data taken from RNA-seq TPM profiles. *p<0.05, ***p<1E-12 calculated using a linear mixed effect model **(F)** or with HOMER **(E)**.

INGAP-PP treatment enhances insulin secretion in response to glucose (**Figure 1B**). However, the peptide effects on β-cells could be direct, through activation of β-cell gene regulatory programs, or indirect, in response to signals elicited from endothelial and/or stellate cells following INGAP-PP treatment. To focus on the effects potentially elicited by INGAP-PP directly on β-cells, we integrated the INGAP-PP regulated genes with epigenomic data profiled in INS-1 cells as a surrogate of rat pancreatic β-cells. For this purpose, we reanalyzed histone modification ChIP-seq and accessible chromatin (ATAC-seq) profiles obtained from untreated INS-1 cells (45). These histone modifications are associated with active (H3K27ac) promoter (H3K4me3) and enhancer (H3K4me1) regulatory regions, while H3K36me3 and H3K79me2 are usually enriched within the body of actively transcribed genes. We also reanalyzed Neurod1 ChIP-seq profiles in untreated INS-1 cells (46). These data were used as a proxy for the rat β-cell epigenomic landscape to further characterize the INGAP-PP-regulated genes. Of note, 1,353 (81%) out of the 1,669 promoters of the INGAP-PP-regulated genes had H3K4me3 signal in INS-1 (**Figure 2A**). Moreover, 1,302 of these (96%) are already active in INS-1 (i.e. regions also enriched in H3K27ac). Thus, most INGAP-PP-regulated genes are active in β-cells.

Increased expression of already active genes can be achieved by cooperative recruitment of enhancers (47). We identified 25,803 enhancers with open chromatin in INS-1, of which 1,362 were associated with the INGAP-PP regulated genes that had H3K4me3 signal in INS-1 (**Figure 2B**). This set of distal genomic regions could indeed be used as a proxy for the study of regulatory events underlying the gene expression changes induced by INGAP-PP in β-cells. We will refer here on to these regions as potential β-cell “INGAP-PP enhancer effectors”.

We next sought to identify the sequences that discriminate the INGAP-PP enhancer effectors from enhancers similarly associated with all genes with H3K4me3 in INS-1 cells. A differential *de novo* motif analysis revealed enrichments for motifs matching STAT2/RUNX1, HIF1, and MEF2 DNA binding sequences, among others (**Supplementary Table 6**). Moreover, an unsupervised k-means clustering of the INGAP-PP enhancer effectors based on the H3K4me1, H3K4me3, and H3K27ac ChIP-seq signal within a 6Kb window centered at the ATAC-seq summits revealed 3 distinct groups that clearly differed in the H3K27ac signal levels (**Figures 2C, D**). These were thus named as active (high), active (low), and poised enhancers (mostly devoid of H3K27ac signal). A differential *de novo* motif analysis performed on these regions revealed DNA sequences selectively enriched in each of these subsets, further pointing to transcription factors potentially underlying the regulatory events triggered by INGAP-PP (**Figure 2E**, **Supplementary Table 7**). The small subset of enhancer regions that is already highly active in untreated INS-1 cells is enriched in E2F motifs, consistent with the high proliferation rate of this β-cell line and with the increased *E2f1* expression trend in rat islets treated with INGAP-PP (**Figure 2F**). Interestingly, active (low) enhancers were selectively enriched in TGIF, RUNX, and HIF1 DNA binding motifs, consistent with activation of TGF-β, Notch, and hypoxia/Vegf signaling in β-cells upon INGAP-PP treatment (**Figures 1C-E**). On the other hand, poised enhancers were differentially enriched in MEF2, NFAT, and VDR motifs (**Figure 2E**, **Supplementary Table 7**). Noteworthy, the expression of genes coding for most of these factors is significantly upregulated in rat islets treated with INGAP-PP (**Figure 2F**), and their epigenomic profiles are consistent with actively transcribed gnes in β-cells (**Supplementary Figure 3**). Taken together, these results suggest that INGAP-PP treatment could potentiate hypoxia, vitamin D, and glucose-responsive signaling mechanisms to increase islet vascularization and improve β-cell secretory response.

### 3.3 INGAP-PP upregulates a subset of genes associated with the protective effects of vitamin D on interleukin 1β-induced β-cell stress

The inflammatory stress associated with IL1β signaling induces β-cell damage and loss of identity (48) and INGAP-PP has been shown to exert a protective effect (18). Through the reanalysis of RNA-seq data from INS-1 cells treated with IL1β or vehicle (49), we found that 47% (637 out of 1,353) of the INGAP-PP-regulated genes with H3K4me3 signal in INS-1 (**Figure 2A** and **Supplementary Figure 4A**) are indeed transcripts suppressed by IL1β in this cell line (*p*=5.3 × 10^-57^, hypergeometric test using 8,898 genes downregulated by IL1β in INS-1). Thus, INGAP-PP would counteract the adverse effects of IL1β-induced cellular stress by upregulating a subset of genes that is negatively modulated by exposure to this cytokine.

**Figure 3:**
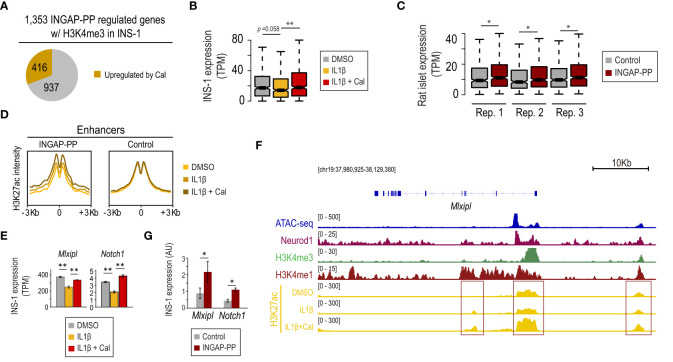
INGAP-PP upregulates a subset of genes associated with the β-cell protective effects of vitamin D against IL1β-induced stress. **(A)** INGAP-PP-regulated genes with H3K4me3 signal in INS-1 overlap with transcripts that are upregulated by the IL1β+calcipotriol (Cal) treatment. **(B, C)** Expression pattern of the genes upregulated by the IL1β+Cal treatment in INS-1 cells in: **(B)** INS-1 cells treated with either IL1β, IL1β+Cal, or control (DMSO), and **(C)** INGAP-PP-treated rat pancreatic islet replicates. The boxes show the IQR of RNA levels, whiskers extend to 1.5 times the IQR or extreme values and notches indicate 95% confidence intervals of the median. **(D)** Aggregation plots showing H3K27ac ChIP-seq signal enrichments, profiled in INS-1 cells treated with either IL1β, IL1β+Cal, or control (DMSO), centered (+/- 3Kb) at the “INGAP-PP enhancer effectors” associated with the gene subset defined as in **(A)**. **(E)** *Mlxipl* and *Notch1* expression is downregulated by IL1β in INS-1 cells, but its expression is restored by the IL1β+ Cal treatment. **(F)** Integrative Genomic Viewer screenshot showing the epigenomic profile at the *Mlxipl* loci. The genomic region visualized is shown in square brackets. Red boxes indicate the gene promoter and potential enhancer regions. **(G)** *Mlxipl* and *Notch1* expression is upregulated in INS-1 by INGAP-PP treatment. RT-qPCR data is normalized to *Actin* gene expression (n=4 biological replicates). Unless otherwise specified, data are expressed as mean ± SEM. *p<0.05, **p<0.01 by Wilcoxon rank-sum test **(B, C)**, EdgeR **(E)** or t-test **(G)**.

Our results suggest that the INGAP-PP effects could be mediated, at least partially, by activation of the vitamin D receptor. Interestingly, it has recently been reported that vitamin D partially counteracts IL1β adverse effects, thus protecting β-cells (49). Indeed, an in-depth analysis revealed that 31% (416 out of 1,353) of the INGAP-PP-regulated genes with H3K4me3 signal in INS-1 (**Figure 2A**) were upregulated by co-treatment of INS-1 with calcipotriol (Cal), a synthetic VDR ligand, on an ILlβ background (*p*=5.3 × 10^-13^, hypergeometric test using 7,277 genes upregulated in INS-1 by ILlβ +Cal *vs* ILlβ alone, **Figures 3A, B**). This subset of genes, which also presented a downregulation trend with the IL1β treatment in INS-1 cells (**Figure 3B**), was significantly upregulated by INGAP-PP in islets (**Figure 3C**).

The transcriptional effects in IL1β+Cal-treated INS-1 cells were correlated with an increase in H3K27ac signal at the β-cell “INGAP-PP enhancer effectors” (**Figure 2B**), suggesting that the Vdr activates these genomic regions (**Figure 3D**). These results are consistent with the enrichment of the VDR motif in a subset of these enhancers (**Figure 2E**). As illustrative examples, we note that *Mlxipl* and *Notch1* (involved in glucose-stimulated β-cell proliferation (50) and protection against stress-induced apoptosis (51), respectively) were downregulated by 30% in IL1β-treated INS-1 cells and its expression was restored to near control levels by co-treatment with Cal (**Figure 3E**). These transcriptional effects are consistent with increased H3K27ac signal at the *Mlxipl* and *Notch1* promoter and putative enhancer regions in INS-1 cells treated with IL1β+Cal, compared to IL1β alone (**Figure 3F**, red boxes). Further supporting our results in rat islets (**Supplementary Figure 4C**), we experimentally validated that *Mlxipl* and *Notch1* are induced by INGAP-PP treatment in INS-1 cells (**Figure 3G**). Taken together, these findings suggest that a subset of genes associated with the protective effects of vitamin D against β-cell stress induced by IL1β is also significantly upregulated in INGAP-PP-treated rat islets.

### 3.4 INGAP-PP induces the expression of transcripts previously unannotated in the rat genome

We next sought to characterize the 75 previously unannotated rat transcripts modulated by INGAP-PP in pancreatic islets. Twelve of them had coding potential, 8 of which were identified as pseudogenes duplicated in the rat genome (**Supplementary Table 8**).

**Figure 4:**
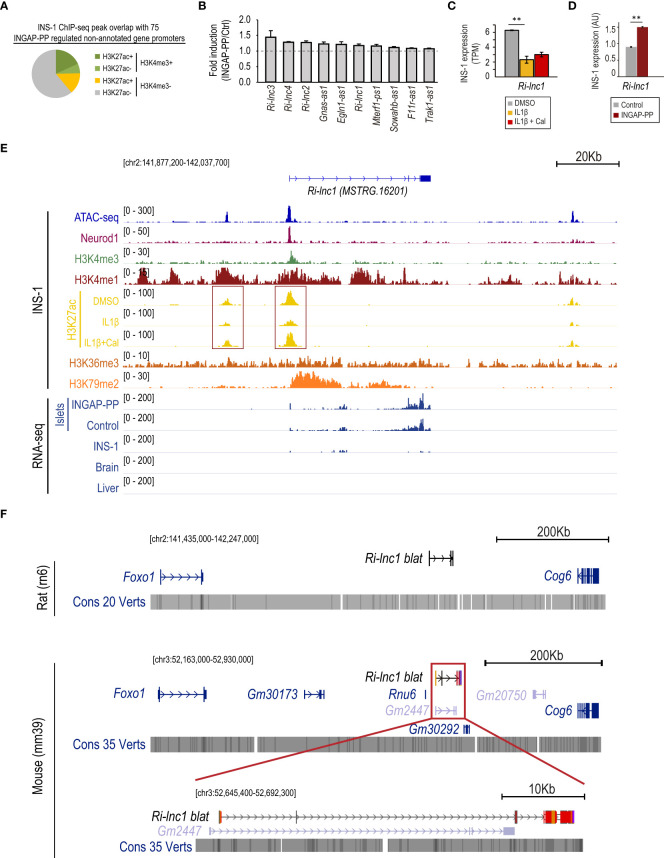
INGAP-PP induces the expression of genes previously unannotated in the rat genome. **(A)** INS-1 H3K4me3 and H3K27ac ChIP-seq peak overlap with the promoters of non-annotated genes regulated by the INGAP-PP treatment in rat pancreatic islets. **(B)** Average gene expression fold enrichment in rat pancreatic islets for selected previously unannotated genes regulated by INGAP-PP. Data taken from RNA-seq TPM profiles. **(C)** *Ri-lnc1* expression is downregulated by IL1β in INS-1 cells, and its expression is not restored by the IL1β+Cal treatment. The data is presented as the average plus standard error of TPM values derived from the duplicate RNA-seq samples analyzed in this study. **(D)** *Ri-lnc1* expression is upregulated in INS-1 cells following INGAP-PP treatment. The RT-qPCR data is normalized to *Actin* gene expression and expressed in arbitrary units (AU), representing the average plus standard error calculated from biological replicates (n=4). **(E)** Integrative Genomic Viewer screenshot showing the epigenomic profile (in untreated INS-1 cells) and the RNA-seq pile-up signal for islet, brain and liver samples analyzed in this study. Most of the non-annotated transcripts with active promoters in INS-1 also show H3K36me3 and H3K79me2 enrichments along the gene bodies, consistently with actively transcribed genes. Red boxes indicate the gene promoter and potential enhancer regions. **(F)** UCSC Genome Browser screenshots of the MSTRG.16201 *(Ri-lnc1)* locus showing the neighbor genes identified in the NCBI Rattus Norvegicus Annotation Release 106 and the NCBI Mus musculus Annotation Release 109. The location of MSTRG.16201, mapped by the Blat tool of the Genome Browser, is shown in black (in contrast to the validated or predicted genes, shown in blue or light blue, respectively). Dark red regions within the MSTRG.16201 blat results projected in the mouse genome indicate non-conserved nucleotide sequences. The genomic regions visualized in panels **(D, E)** are shown in square brackets. Unless otherwise specified, data are expressed as mean ± SEM. **p<0.01 by EdgeR **(C)** or t-test **(D)**.

An in-depth epigenomic analysis revealed that the promoter region of 19 out of the 75 unannotated transcripts overlapped with an H3K4me3 peak in INS-1 cells. Ten of them also had promoters additionally overlapping H3K27ac peaks and were expressed in INS-1 cells above the detection threshold, thus suggesting that these are active genes in β-cells (**Figures 4A, B**, **Supplementary Table 8**). Only one of them could be annotated as a partial duplication of *Mterf1*. The 9 remaining genes were not previously annotated in the rat genome, could not be identified with any previously annotated nucleotide sequence in the NCBI and did not map to any annotated gene at the homologous location in the mouse or human genomes. Importantly, the transcripts associated with these genes were longer than 200 bp and were not found to have coding potential. Thus, we identified 9 novel lncRNAs that: 1) are expressed above the detection threshold in rat pancreatic islets, 2) have active promoters in untreated INS-1 cells, and 3) are induced in INGAP-PP treated islets. Five of them mapped in antisense to already annotated protein-coding genes and were renamed as “antisense” (-as) followed by their gene names (e.g. *Sowahb-as1*, **Supplementary Figures 5A-E**). The remaining lncRNAs were renamed as *Ri-lnc1-4* after Rat islet long non-coding RNA 1 to 4.

Consistent with the findings described above, 4 of the 9 novel lncRNAs reported are downregulated in IL1β-treated INS-1 cells and *Ri-lnc2* expression is upregulated by co-treatment with Cal (**Supplementary Figures 5A-E**). In particular, *Ri-lnc1* expression is severely suppressed by IL1β in INS-1 cells (**Figure 4C**) and, in agreement, the H3K27ac signal at its promoter and a putative enhancer region is decreased upon IL1β exposure (**Figure 4E**, red boxes). Further supporting our results in rat islets, we experimentally validated that *Ri-lnc1* is induced by INGAP-PP treatment in INS-1 cells (**Figure 4D**). Importantly, the gene encoding for *Ri-lnc1* has active promoter marks and a strong Neurod1 binding site at its promoter, thus suggesting a relevant function in β-cells (**Figure 4E**). The closest genes to *Ri-lnc1* are *Foxo1* (upstream) and *Cog6* (downstream), both located more than 200 Kb away. Of note, *Ri-lnc1* does not overlap with other genes and it is not located antisense to other annotated transcripts in the rat genome (**Figure 4F**, top). In the mouse genome, it partially overlapped the predicted gene *Gm2447*, but a detailed inspection revealed that the exon nucleotide sequences are largely non-conserved (**Figure 4F**, bottom). No overlaps were found in the human genome (**Supplementary Figure 6**).

Interestingly, the putative promoter regions for 16 out of the 75 unannotated transcripts that lacked H3K4me3 enrichment, overlapped with H3K4me1 peaks in INS-1. Eleven of them also overlapped with H3K27ac peaks, thus suggesting that these could be transcribed enhancer regions (52). An illustrative example is shown in **Supplementary Figure 7** for MSTRG.4242.

### 3.5 *Ri-lnc1* is associated with the subset of mature β-cells

Finally, we evaluated the relevance of our novel gene annotation for the analysis of transcriptomic data at the single-cell level. For this purpose, we adapted the *de novo* rat genome annotation reported here for its use with Cellranger and reanalyzed a very recently published rat pancreatic islet single-cell RNA-seq dataset (37) consisting of islets exposed to high glucose and treated with oleate, palmitate or the vehicle.

**Figure 5:**
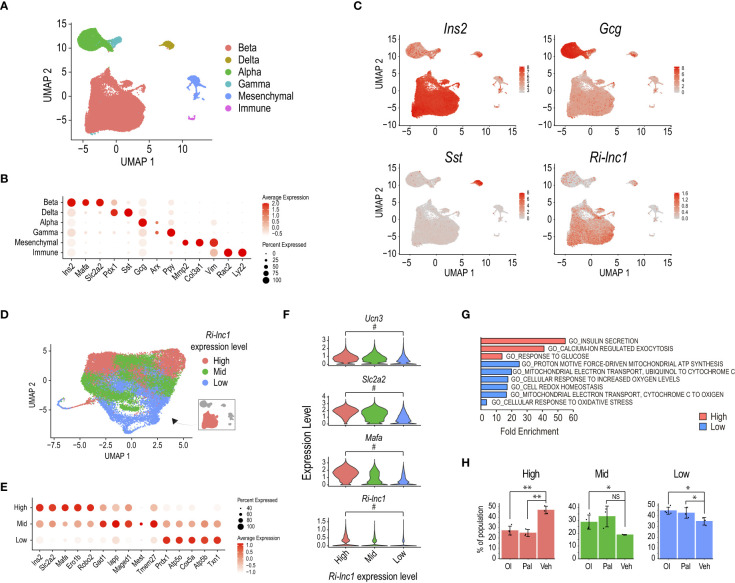
*Ri-lnc1* is associated with the subset of mature β-cells. **(A)** UMAP plot of 37,769 single cell transcriptomes profiled from oleate (Ol), palmitate (Pal) or vehicle (Veh) treated rat pancreatic islets (37). Colors in the UMAP highlight clustering into the main islet cell subtypes. **(B)** Dot plot showing the expression of key endocrine, mesenchymal or immune cell type markers used to name clusters in **(A)**. Color intensity indicates mean expression (normalized) in a cluster, and dot size indicates the proportion of cells in a cluster expressing the gene. **(C)** Feature plots showing enriched expression of *Ins2*, *Gcg* and *Sst* in the β, α and δ cell clusters, respectively. *Ri-lnc1* is also enriched in the β-cell cluster. **(D)** UMAP plot of 28,894 single-cell transcriptomes taken from the β-cell subset in **(A)**. Colors in the UMAP highlight clustering into 3 different levels of *Ri-lnc1* expression. **(E)** Dot plot showing the expression of selected markers enriched in each cluster. Color intensity indicates mean expression (normalized) in a cluster, dot size indicates the proportion of cells in a cluster expressing the gene. **(F)** Violin plots showing expression for well-known mature β-cell markers *Ucn3*, *Slc2a2* and *Mafa*, as well as *Ri-lnc1*, in β-cells clustered as in **(D)**. # indicates that this gene is a differential marker between the *Ri-lnc1* High and Low clusters. **(G)** Functional annotation of the marker genes for *Ri-lnc1* high and low range expression clusters. **(H)** Percentage of each β-cell cluster over the total β-cells for each islet treatment condition. *p<0.05, **p<0.01, calculated by t-test. NS, not significant.

The unbiased graph-based clustering of cells pooled from all three culture conditions identified the four main endocrine cell types based on the expression of *Ins2*, *Gcg*, *Sst*, and *Ppy*, corresponding to β, α, δ and γ cells, respectively (**Figures 5A, B**). We also detected non-endocrine cells expressing *Col3a1* and *Vim* (mesenchymal cells) or *Rac2* and *Lyz2* (immune cells). Importantly, this unbiased analysis specifically retrieved *Ri-lnc1* as a marker of the β-cell cluster, consistent with its β-cell selective expression pattern (**Figure 5C**). Five of the other lncRNAs reported here presented a pan-endocrine expression pattern, and MSTRG.4242 (potentially a transcribed enhancer) expression was highly enriched in β-cells (**Supplementary Figure 8A**).

Refined clustering of cells in the Ins2+ cluster identified several β-cell subpopulations which presented varying expression levels for *Ins2*, *Slc2a2* (coding for Glut2) and *Mafa* (**Supplementary Figures 8B-D**). Of note, *Ri-lnc1* was also expressed at different levels among these clusters, suggesting that it could be associated with specific β-cell subsets. To gain further insights into the potential role of this lncRNA, we grouped clusters based on the level of *Ri-lnc1* expression. We thus defined 3 broader clusters accounting for *Ri-lnc1* High (9,061 cells), Mid (11,941 cells) or Low (7,892 cells) range expression (**Figure 5D**). Based on the expression level of *Ucn3*, *Slc2a2*, and *Mafa*, these clusters matched mature (*Ri-lnc1* High), intermediate (*Ri-lnc1* Mid) and immature (*Ri-lnc1* Low) β-cells (**Figures 5E, F**). Thus, *Ri-lnc1* expression was tightly correlated to these well-known markers. Also, β-cells in the *Ri-lnc1* Low cluster expressed genes involved in oxidative phosphorylation and oxidoreductase activity–related pathways (e.g. *Txn1*, *Atp5b* and *Cox5a*, **Figures 5E, G**). It has been recently reported that rat pancreatic islet exposure to fatty acids (oleate, in particular) amplified oxidative phosphorylation and antioxidant pathways, diminishing β-cell maturation (37). Accordingly, we observed that the number of cells in *Ri-lnc1* High cluster (accounting for mature β-cells) was lower for both oleate and palmitate treatments, when compared to the vehicle (**Figure 5H**). Conversely, the number of cells in the *Ri-lnc1* Low cluster (containing β-cells with high expression of genes associated with oxidative phosphorylation and antioxidant pathways) was larger for both treatments. Taken together, these results suggest that *Ri-lnc1* is a novel marker for mature β-cells and further support that our new annotation may help to better characterize the transcriptomic outcomes of rat pancreatic islets exposed to a variety of stimuli. Further research is warranted to assess the specific roles that these transcripts play in β-cell function.

## 4 Discussion

Considering the substantial body of evidence already published regarding the enhancing effect of INGAP-PP on β-cell function and mass in *in vivo* and *ex vivo* animal models, along with promising results from clinical trials (such as reduced HbA1c levels in T2D and increased C-peptide in T1D), we hypothesize that INGAP-PP or a more tolerable analog holds potential as a tool for diabetes treatment. Consequently, we describe here the global transcriptional changes elicited by INGAP-PP *in vitro* treatment in cultured rat pancreatic islets based on a *de novo* annotation of the rat genome. We found 1,669 differentially regulated genes, including many previously unannotated rat transcripts, most of which are upregulated. These are expected to, at least partially, explain the improvement in insulin secretion after peptide treatment (**Figure 1B**). Noteworthy, here we aimed at characterizing the INGAP-PP transcriptional effects in whole pancreatic islets as a micro-organ, therefore we profiled them as a population. This approach prioritized capturing a larger number of genes with greater precision at the expense of detecting heterogeneity of gene expression in islet cells at the single-cell level (53). Future single-cell RNAseq studies are warranted to explore the INGAP-PP effects on individual islet cell types.

A functional annotation of this set of genes revealed enrichments for known (PI3K, ERK, VEGF) and potentially novel (mTOR, GPCR) signaling pathways regulated by INGAP, which play important roles in β-cell function or regeneration (11, 17, 19, 33, 43, 54, 55). Our results also suggest that INGAP-PP treatment may be involved in the activation of pancreatic stellate cells, which could potentially play a role in tissue regeneration, similar to what has been reported for their liver counterparts (56).

Using INS-1 cells as a surrogate for β-cells, we demonstrate that most of the genes regulated by INGAP-PP have active promoters even in untreated cells. Therefore, it is likely that INGAP-PP enhances β-cell function by increasing the expression of genes that are already active. A *de novo* motif analysis in β-cell enhancers, potentially regulated by INGAP-PP to achieve its function, suggests that treatment with this peptide may involve hypoxia and glucose-responsive signaling mechanisms. These mechanisms have previously been demonstrated to enhance the intra-islet angiogenesis process and improve insulin secretion response (20). Specifically, our analysis revealed that active (low) regulatory regions in INS-1 cells were enriched in HIF1 motifs, while poised enhancers were enriched in NFAT and VDR motifs (**Figure 2E**). Our results suggest that the coordinated activation of these factors could underlie the transcriptional effects induced by INGAP-PP.

Earlier studies have demonstrated the essential role of ARNT/HIF1β and HIF1α in glucose-stimulated insulin release (57, 58). Similarly, NFAT factors are well-known regulators of insulin expression and secretion, subject to dynamic regulation through ERK1/2 activation in response to glucose and calcium signaling (59, 60). Consequently, the enrichment of NFAT motifs in INS-1 poised enhancers associated with genes regulated by INGAP-PP aligns with the reported influence of INGAP-PP on ERK1/2 activation (54). This could potentially explain, at least in part, the observed improvement in insulin secretion in INGAP-PP-treated rat islets (**Figure 1B**).

Notably, cell treatment with vitamin D (and thus VDR activation), promotes β-cell survival and enhances pancreatic insulin synthesis and secretion in response to high glucose levels, both *in vivo* and *in vitro* (49, 61, 62). Considering these findings alongside our GSEA results (**Figure 1G**), it is plausible that INGAP-PP may activate the vitamin D signaling pathway. Further reinforcing this hypothesis, we found that a subset of genes associated with the protective effects of vitamin D against IL1β-induced β-cell stress is significantly upregulated upon INGAP-PP treatment of rat islets. Collectively, these findings suggest that enhancing INGAP-PP-based treatment formulations for people with diabetes could potentially involve co-treatment with vitamin D receptor agonists.

While integrating the results obtained from primary rat pancreatic islets with epigenomic and transcriptomic profiles obtained from INS-1 cells provided essential support for some of our findings (e.g., the identification and regulation of novel lncRNAs), we acknowledge that this comparison presents a limitation in the current analysis. Importantly, rat β-cells exhibit limited cell division, whereas INS-1 cells possess an active replication machinery. This distinction is underscored in our results, where we observe that a small subset of enhancers associated with INGAP-PP-regulated genes is already highly active in untreated INS-1 cells (**Figures 2D, E**). This suggests that while the INGAP-PP effects on rat β-cells might involve enhancing cell proliferation through the activation of these regions, the impact of the peptide on INS-1 cells in these regions might not be as pronounced. Consequently, the biological significance of the mechanisms discussed above for *in vivo* rat pancreatic β-cells remains to be established.

Finally, we report 9 novel long non-coding transcripts previously unannotated in the rat genome that are upregulated by INGAP-PP, some of which are downregulated in INS-1 cells by IL1β treatment. To facilitate their future characterization, we have included them in the NCBI nucleotide database. As a proof of concept, a reanalysis of recently published scRNA-seq data using the novel genome annotation revealed that *Ri-lnc1* expression levels vary among the different β-cell clusters. It is most often co-expressed with mature β-cell markers such as *Mafa*, *Slc2a2* and *Ucn3*, and it is significantly downregulated in β-cells with activated oxidative phosphorylation and antioxidant pathways.

It should be noted that, like most lncRNAs, *Ri-lnc1* is not conserved at the sequence level in either mice or humans, even though it presents a partial overlap with a predicted gene in mice (*Gm2447*, **Figure 4F**). We did not find any annotated lncRNA overlapping *Ri-lnc1* in the human genome (**Figure S6**, **Table S8**). However, despite lncRNAs displaying low sequence conservation across species compared to protein-coding genes, their functions can be conserved or functionally analogous (63). Further investigation is warranted into the conservation of function in other species for some of the newly annotated rat lncRNAs discussed here. This aspect holds particular significance as it might help elucidate limitations in translating these results to humans.

Collectively, our results unveil the underlying selective transcriptional program that could at least partially explain the enhancement of INGAP-PP-mediated physiological effects on β-cell mass and function. The findings presented in this study are expected to serve as the foundation for a deeper comprehension of islet translational outcomes from rodents to humans, ultimately contributing to the design of improved therapies for individuals with diabetes through the administration of INGAP-PP or one of its analog peptides.

## Data availability statement

The datasets presented in this study can be found in online repositories. The names of the repository/repositories and accession number(s) can be found below: https://www.ncbi.nlm.nih.gov/geo/, GSE208002, https://www.ncbi.nlm.nih.gov/genbank/, OQ067272 and OQ729945-53.

## Ethics statement

The animal study was approved by the Animal Welfare Committe (CICUAL) of La Plata School of Medicine, UNLP (T01-04-2021). The study was conducted in accordance with the local legislation and institutional requirements.

## Author contributions

JG, BM, LF and SR-S conceptualized the work and designed the experiments. CR, BM and LF generated RNA biological samples for control and INGAP-PP treatments and performed insulin secretion validations. AH performed RNA-seq alignments, rat *de novo* transcriptome annotation and quantification, and scRNA-seq reanalysis. AR, AH, MA and SR-S performed downstream transcriptomic and epigenomic analyses. AR and SR-S performed *de novo* motif analyses. AR, MA, CR, EN, BM and LF performed RT-qPCR validations. AR and SR-S wrote the manuscript, with contributions from all authors. All authors discussed the results, read and approved the final manuscript version. SR-S is the guarantor of this work and, as such, had full access to all the data in the study and takes responsibility for the integrity of the data and the accuracy of the data analysis.
