# Supplementary material for: Transcriptional signature of islet neogenesis-associated protein peptide-treated rat pancreatic islets reveals induction of novel long non-coding RNAs
Source: Front Endocrinol (Lausanne). 2023 Sep 29;14:1226615. doi: 10.3389/fendo.2023.1226615 (PMC10570750; doi:10.3389/fendo.2023.1226615)
Supplement: Supplementary file 1 [file DataSheet_1.docx]

Supplementary Material

**Transcriptional signature of islet neogenesis-associated protein peptide-treated rat pancreatic islets reveals induction of novel long non-coding RNAs**

**Authors**

Agustín Romero,^1,2†^ Ana C. Heidenreich,^1,2†^ Carolina L. Román,^3^ Macarena Algañarás,^3^ Ezequiel Nazer^1^, Juan J. Gagliardino,^3^ Bárbara Maiztegui,^3^ Luis E. Flores,^3^ Santiago A. Rodríguez-Seguí^1,2*^

^†^ These authors contributed equally to this work and share the first authorship

^*^ Corresponding author: Santiago A. Rodríguez-Seguí, [srodriguez@fbmc.fcen.uba.ar](mailto:srodriguez@fbmc.fcen.uba.ar)

**Supplementary methods**

**Animals and islet isolation.** Adult male Wistar rats (body weight, 230–260 g) were maintained under controlled conditions of 23°C and a fixed 12-hour light, 12-hour dark cycle (6:00 A.M.–8:00 P.M.), with free access to a standard commercial diet and water. Experiments were performed according to the “Ethical principles and guidelines for experimental animals” (3rd ed., 2005) from the Swiss Academy of Medical Sciences. Animals were euthanized by cervical dislocation, and the animal study was reviewed and approved by Animal Welfare Committe (CICUAL) of La Plata School of Medicine, UNLP ( T01-04-2021). At the time of euthanasia, the whole pancreas from each animal was removed to isolate islets by collagenase digestion (1).

**Islet culture.** Freshly isolated islets were cultured in RPMI-1640 media (Gibco BRL, Carlsbad, CA) pH 7.4, containing 2 g/L NaHCO_3_, 5% (v/v) fetal bovine serum, 1% penicillin/streptomycin, and 10 mM glucose at 37°C in a humid atmosphere (5% CO_2_/95% O_2_). Islets were cultured for 4 days in the absence (C, control) or presence of 50 μg/mL INGAP-PP. The medium was renewed every other day. Thereafter, islets were preincubated in Krebs-Ringer bicarbonate (KRB) buffer, pH 7.4, previously gassed with a mixture of CO_2_/O_2_ (5/95%), containing 1% (w/v) BSA and 3.3 mM glucose at 37°C for 45 minutes. The INGAP-PP (NH-Ile-Gly-Leu-His-Asp-Pro-Ser-His-Gly-Thr-Leu-Pro-Asn-Gly-Ser-COOH) was kindly provided by Dr. G. Alexander Fleming (Kinexum LLC, Harper's Ferry, West Virginia) or purchased from GL Biochem (Shanghai, China). Quality control of the peptide (amino acid analysis and mass spectrometry) indicated greater than 95% purity and a molecular weight of 1501.63.

**INS-1 cell culture and treatment.** INS-1 cells were cultured in RPMI-1640 media (Gibco BRL, Carlsbad, CA) pH 7.4, containing 2 g/L NaHCO_3_, 10% (v/v) fetal bovine serum, 1% penicillin/streptomycin and 50µM of 2-mercaptoethanol, at 37°C in a humid atmosphere (5% CO_2_/95% O_2_). Cells were treated for 48h in the absence (C, control) or presence of 40 μg/mL INGAP-PP.

**Insulin Secretion.** Groups of 5 islets from both groups were incubated for 60 minutes in 0.6 mL KRB with different glucose concentration (3.3 or 16.7 mM). At the end of the incubation period, aliquots of the medium were collected for insulin determination by radioimmunoassay. Three independent measurements were made for each treatment replicate using medium samples obtained for different islet aliquots. For this procedure, we used an antibody against rat insulin (Sigma Chemical Co.) and rat insulin standard (Novo Nordisk Pharma Argentina) and highly purified porcine insulin labeled with ^125^I (2). To normalize insulin measurements, we also quantified the islet DNA content in islets from each experimental group. For this purpose, islets were homogenized in TNE buffer and stored at −70°C for subsequent measurement by a fluorometric assay.

**RNA isolation and RT-qPCR.** Total RNA was isolated using the RNeasy Mini Kit (Qiagen, #74106) and eluted in 30 μl of RNase free water. RNA integrity was tested by agarose-formaldehyde gel electrophoresis. Possible contamination with protein or phenol was controlled by measuring the 260:280 nm absorbance ratio, while DNA contamination was avoided by treating the sample with DNase I (Invitrogen). One µg of total RNA was used for cDNA synthesis with SuperScript III Reverse Transcriptase (Invitrogen, #18064014). Quantitative Real-Time PCR was performed using the FastStart SYBR Green Master (Roche) on the iCycler 5 Real-Time PCR system (BioRad) according to the manufacturer’s instructions. Samples were run in technical triplicates and normalized to β-actin. Gene-specific primers are listed in **Supplementary Table 9**.

**RNA sequencing.** RNA from triplicate INGAP-PP and untreated samples (10µg at concentration ≥80 ng/µl) was assessed for quality by Agilent bioanalyzer prior to library generation and sequencing. All samples had RNA integrity number (RIN) > 9. Paired-end RNA-seq libraries were sequenced on Illumina HiSeq4000, obtaining >92 million 100bp reads.

**RNA-seq alignment and gene expression quantification.** In order to get a more accurate quantification of gene expression, we applied the HISAT-StringTie analysis pipeline (3) for *de novo* annotation of genes expressed in rat tissues. To further improve the precision of the gene structure detection of rat transcripts, our triplicate RNA-seq datasets for INGAP-PP treated and control islets were analyzed together with a series of different rat tissue RNA-seq datasets consisting of brain, liver and pancreatic islet samples obtained from public databases (**Supplementary Table 1**). A combined analysis pipeline using this resource allowed us to generate an improved annotation of the rat genome which better matched read alignment profiles as revealed by the RNA-seq signal detected in each tissue. This approach, run on a series of different rat tissue RNA-seq samples, allowed curating the current rat genome reference annotation (Rnor_6.0, downloaded from <https://www.ncbi.nlm.nih.gov/assembly/GCF_000001895.5/>) which was used as input to the pipeline.

To improve gene structure detection for all rat transcripts expressed in the samples analyzed, we aimed at increasing read coverage by merging all raw sequence (fastq) files from all replicates of each particular sample. Thus, we generated 4 “merged” fastq files corresponding to each of the 4 samples analyzed: liver, brain, islets (public datasets) and islets (our samples) with 132752218, 152431871, 114806764 and 257356155 total reads, respectively. These files were aligned to the UCSC rn6 rat genome (<https://genome-idx.s3.amazonaws.com/hisat/rn6_genome.tar.gz>) using HISAT with default settings. This retrieved a read alignment file that was feeded to the StringTie pipeline (3). In brief, running the StringTie command generated an “Assembled transcripts” file, which was then merged with the original reference genome annotation used (Rnor_6.0) to generate a “Merged transcripts” file including both correctly annotated genes in the original reference, as well as *de novo* annotated genes and variants detected by the HISAT-StringTie pipeline. This “curated rat reference genome annotation” was used in all the following analyses.

HISAT was next used to align raw sequence reads from each sample and replicate separately, generating individual “read alignment” files. The final gene expression quantification for each sample was achieved by using these files, and our newly curated rat genome annotation, as input to the last StringTie command in the pipeline (StringTie -eB) using default settings. The output of this pipeline allowed producing 15 read coverage tables which accounted for gene expression in each of the 6 rat islet samples generated in this study (3 untreated and 3 INGAP-PP treated samples) and 3 replicates for each of the public rat tissue samples included in our analysis (i.e. islets, liver and brain). Individual RNA-seq “read alignment” files were generated for additional samples (**Supplementary Table 1**) at this stage. TPM gene expression tables were used in downstream analysis as described next. Given that read coverage for individual islet samples was not enough (i.e. < 100 million reads per sample) to accurately detect gene splicing variants, transcript variants were collapsed by the StringTie “gene_id”. This resulted in a final list of 32,654 genes, including both previously annotated and new genes. These included 14,701 islet expressed genes, considering a threshold of 0.5 TPM in either all INGAP-PP treated samples, for genes upregulated with the peptide treatment, or in all control samples for downregulated genes.

**Detection of differentially expressed genes in response to the INGAP-PP treatment.** The differential expression of genes was determined by modeling the INGAP-PP effect with a linear mixed effect (LME) model to account for the random effects of different batches of rat donors. Statistical significance of differentially expressed genes (DGEs) were obtained from slope p-value as previously described (4). The set of 1,669 INGAP-PP regulated genes was next defined as genes with LME p-value < 0.05, that are expressed either in Control or INGAP-PP samples above the detection threshold (set to 0.5 TPM), and with an average gene expression change higher or equal than 5% upon INGAP-PP treatment (Avg FC>=1.05 or Avg FC<=0.95).

**Clustering and Principal Component Analyses (PCA).** For the PCA and clustering of samples (**Supplementary Figure 1A**,**B**) we used all genes that were expressed >1 TPM in all replicates of at least one of the 5 set of samples analyzed (i.e. liver, brain and islet public datasets, and control and INGAP-PP-treated islets). This reduced the total number of genes originally quantified by the HISAT-StringTie pipeline from 32,654 (which included non-expressed genes) to 17,934, and allowed focusing the analysis on genes consistently detected above a more stringent threshold set (1 TPM) in at least one sample. The Log2 of the expression values (TPM+1) was calculated for all genes in the reduced table and PCA and clustering were performed using R (PCA, hclust functions).

**Gene Ontology Analysis (GO).** GO was performed using panther (<http://geneontology.org/>) with only 1,669 up-regulated genes in INGAP-PP treatment, of which 1,383 were recognized by panther, the cause of this exclusion of 285 genes was because this were no annotated genes or no affiliated to a predicted pathway. Significantly enriched pathways (FDR<0.05; *p*<0.05) are presented in **Supplementary Table 4.** The most interesting pathways with a significant FDR and the highest fold enrichment scores are presented in the Circus plot (5) of **Figure 1D**.

**Gene Set Enrichment Analysis (GSEA)**. GSEA v3.0 (6) was performed with default parameters using the list of annotated INGAP-PP regulated genes (1,593 annotated genes, out of 1,669 total genes, **Supplementary Table 3**). For this purpose, we pre-ranked the list of genes by the averaged log2 fold-change replicate expression and interrogated them with the canonical pathways and gene ontology (biological process) gene sets annotated from the MsigDb database. Significantly enriched genesets (*p*<0.05 and FDR<0.25) in any of the pairwise comparisons are presented in **Supplementary Table 5**.

**Single-cell RNA-seq.** Single-cell RNA-seq data from dissociated mouse pancreatic islets was taken from Baron et al. (7). Already quantified and cell-type clustered data was downloaded from the Gene Expression Omnibus (GEO) with accession GSE84133. Data was processed for visualization of violin plots to show the expression of selected genes using the ScanPy package (8). Single-cell RNA-seq data from dissociated rat pancreatic islets was taken from Vivoli et al. (9). Raw data was downloaded from the Gene Expression Omnibus (GEO) with accession GSE193857. We next built a custom genome reference, based on the “curated rat reference genome annotation” created in this work, using the CellRanger (v.7.1.0) *mkref* function following the steps described in the 10xgenomics webpage for this purpose. Reads from .*fastq* files were aligned using Cellranger *count* function using the “curated rat reference genome annotation” as transcriptome reference input file. Data from triplicate vehicle and palmitate samples, and quadruplicate oleate samples was re-quantified with Cellranger as described above. Downstream analysis were performed with Seurat (v.4.0.4), these included single cell filtering (nFeature between 2000 and 5000, nCount between 5000 and 50000 and mitochondrial percentage lower than 10) integration parameters were set to 30 for npcs, 3000 highly variable features were selected and n-neighbors were set to 20. For cluster detection and filtering of non-endocrine clusters, resolution parameter was set to 0.4. We next selected the β-cells subset for in depth analysis, excluding a small β-cell subcluster expressing high levels of *Gcg* and *Ins2*. The remaining β-cells were reanalyzed using 3000 highly variable features for data integration, npcs=30, and the Seurat *FindNeighbors* and *FindClusters* functions with default parameters. The resulting clusters were next merged according to their average expression of *Ri-lnc1* to obtain 3 new clusters containing cells with high, medium or low *Ri-lnc1* expression level. To define such clusters the upper threshold value was set on 0.3 and lower threshold value was set on 0.18.

**ChIP-seq.** Publicly available raw datasets were obtained from the Sequence Read Archive (SRA) database as listed in **Supplementary Table 1**. Sequence reads were realigned to the rat (rn6) genome using Bowtie v1.3.0 (10), and further processed as previously described (11, 12). Only sequences uniquely aligned with ≤1 mismatch were retained. Processed bam files were visualized in the IGV browser (13). Transcription factor enrichment sites were detected with MACS3 v. 3.0.0a7 (14) using default parameters and a q value cutoff of 0.05. Histone modification islands were called using SICER2 v.1.0.3 using the following parameters. For H3K4me1: -w 100 -egf 0.74 -fdr 0.01 -g 800 -e 1000; and for H3K4me3 and H3K27ac: -w 100 -egf 0.74 -fdr 0.01 -g 200 -e 1000. Human islet transcription factor and histone modification ChIP-seq samples processed as described before (15).

**ATAC-seq.** Publicly available raw datasets were obtained from the Sequence Read Archive (SRA) database as listed in **Supplementary Table 1**. Sequence reads were realigned to the rat (rn6) genome using Bowtie2 v2.3.4.3 (16) using the following settings: --very-sensitive -X 2000. Processed bam files were visualized in the IGV browser (13). ATAC-seq enrichment sites were detected with MACS3 v. 3.0.0a7 (14) with the following parameters: --nomodel --shift -100 --extsize 200 -q 0.05.

**Promoter and enhancer identification.** Global gene promoters were defined as genomic regions +2 Kb/ -1 Kb from the transcription start site (TSS) of all transcripts annotated by the HISAT/StringTie pipeline described above. INS-1 promoter regions were next defined as those global gene promoters that overlapped with INS-1 H3K4me3 islands. Active INS-1 promoters, were defined as those INS-1 promoter regions that additionally overlapped with INS-1 H3K27ac islands. Intersection of these subsets with the promoters of the INGAP-PP regulated genes revealed genes potentially regulated in β cells, using the INS-1 cell line as surrogate.

Enhancer regions in INS-1 were defined as H3K4me1 islands that did not overlap H3K4me3 regions. To improve the accuracy of downstream analyses, we restricted the set of enhancer regions to those overlapping ATAC-seq peaks in INS-1. This resulted in the identification of 25,803 enhancers with open chromatin in INS-1, that were defined by the INS-1 ATAC-seq peak genomic coordinates. Enhancers were next associated to potential gene targets following single nearest gene rule, using the closest gene TSS up to a maximum distance of 1Mbp. Selecting the subset of enhancers associated with the INGAP-PP regulated genes revealed a total of 1,362 regions that were defined as potential β cell “INGAP-PP enhancer effectors”. Region overlap and associations were computed using the Bedtools (v2.30.0) intersectBed, subtractBed and closestBed commands (17). We considered peaks to be overlapping if they shared a minimum of one base.

**Heatmap and aggregation plots.** To compute the heatmap and aggregation (averageogram) plots we divided the bed file containing the regions defined as INGAP-PP enhancer effectors (spanning +/- 3 Kb from the center of the ATAC-seq peaks) in 100 bp bins (**Figure 2C**,**D**). The coverage signal from the H3K4me3, H3K4me1 and H3K27ac INS-1 ChIP-seq aligned read bed files was obtained for each bin using the coverageBed command from BedTools (17). For quantile normalization of the results, we produced 100 bp bins across all the islands called in each sample and calculated the signal coverage of the ChIP-seq dataset in the same sample in which the islands were called. The 95th quantile obtained from the binned peaks in each ChIP-seq sample was used to adjust the bin values obtained for the aggregation plots. The unsupervised k-means clustering of the INGAP-PP enhancer effectors was performed with Cluster3 software (18), setting the number of clusters k=3, based on the H3K4me1, H3K4me3 and H3K27ac ChIP-seq signal at the binned enhancer regions as described above. The heatmaps were produced with Cluster3 software (18) and plotted using Treeview (19).

**Motif Discovery.** *De novo* motif discovery was performed with HOMER (20) using a window of 500 bp centered at the INGAP-PP enhancer effectors (defined by the ATAC-seq peak coordinates as described above) and setting motifs length to 8, 10 and 12 bp. To study the differential motif enrichments within the regions of interest, two complementary analyses were performed using different sets of background regions.

One hand, to identify the *de novo* transcription factor motifs enriched at the potential INGAP-PP enhancer effectors we performed a differential analysis using as background (-bg option in the findMotifsGenome.pl command) all enhancers similarly associated to all genes with H3K4me3 in INS-1 cells (**Supplementary Table 6**). This background file consisted in 13,205 enhancers with open chromatin in INS-1 effectors (defined by the ATAC-seq peak coordinates as described above) that additionally were associated to a closest TSS with a H3K4me3 island in INS-1.

On the other hand, a more detailed *de novo* motif analysis was performed on the different region subsets identified within the INGAP-PP enhancer effectors (**Figure 2E**, **Supplementary Table 7**). To identify the *de novo* transcription factor motifs enriched at the Active (High), Active (Low) and poised INGAP-PP enhancer effectors we performed a differential analysis using as background (-bg option in the findMotifsGenome.pl command) the all INGAP-PP enhancer effectors subsets. The small subset of enhancer regions that is already highly active in untreated INS-1 cells is enriched in E2F motifs, consistent with the high proliferation rate of this β-cell line.

In all cases, the top scoring motifs ranked by P-value (setting a significant threshold of P-value ˂ 0.05) are shown (**Tables S6** and **S7**). Matching DNA binding motifs were associated to the *de novo* recovered matrix when the HOMER score was 0.7 or higher. Only transcription factors with its coding gene expressed above the selected threshold (>0.5 TPM in either Control or INGAP-PP treated samples) are shown.

**Genes regulated by IL1β with or without calcipotriol treatment.** Data from Wei et al. (21) was obtained from the SRA database (**Supplementary Table 1**), realigned to the rat genome, and gene expression was requantified using our curated rat reference genome annotation as described above. To obtain the set of 1,353 of the INGAP-PP regulated genes with H3K4me3 signal in INS-1 that is additionally downregulated by IL1β treatment of INS-1 cells (**Supplementary Figure 4**) we considered a fold change < 0.90 between the IL1β and DMSO expression levels. To obtain the set of these genes that is upregulated by the combined treatment with calcipotriol, when compared to IL1 treatment alone, we considered a fold change > 1.1 between the Cal+IL1 and IL1β expression levels (**Figure 3A**).

**Characterization of rat unannotated genes and homolog gene search**. To characterize genes previously unannotated in the rat genome, we first used its genomic position (as retrieved by our HISAT/StringTie pipeline) to map them to the rat genome (rn6 version) in the UCSC Genome Browser. We validated in this way that no known rat transcripts were annotated in the analyzed regions. To find potential mouse and human homologs we performed a lift over of the gene coordinates from the rn6 rat coordinates to the mm39 (mouse) or hg38 (human) genomes and checked whether any known mouse or human transcript is annotated in the lifted over region using the UCSC Genome Browser. We next double checked that the new annotated sequence (i.e. the RNA sequence of the MSTRG transcript *de novo* aligned to the rat genome using the HiSAT/Stringtie pipeline) mapped to the same region as found in the lift over analyses using the NCBI Blat tool (22), and that it actually overlapped any of the already annotated transcripts in the mouse or human genomes. For cases where we found overlapping genes that met both conditions (even if not completely matching the start and end gene coordinates), we performed an in-depth sequence analysis using the NCBI Blast tool (23). We evaluated in this way if mapping of exon sequences matched between the rat, mouse and human genomes (**Supplementary Table 8**). Complementarily, we evaluated the Coding Potential of each non-annotated transcript using CPC2 (24). Coding probability was calculated using Fickett score, Isoelectric point and ORG integrity as previously described (24).

**Supplementary Figure legends**

**Supplementary Figure 1. Global analysis of the gene expression profiles across a panel of rat tissues.** (A) PCA of the RNA-seq data clearly separates brain, liver and pancreatic islet replicate samples. The barplot below depicts the percent of variance explained by each of the principal components (PC). (B) Heatmap depicting 17,934 genes expressed >1 TPM in all replicates of same tissue/condition. Column-hierarchical clustering groups replicates by tissue of origin, as expected, but reveals that pancreatic islet samples were first clustered by replicate, rather than by control and INGAP-PP treatment. An unsupervised hierarchical clustering grouped each INGAP-PP treated sample closer to its corresponding control than to other pentadecapeptide-treated samples. Our replicate islet samples were next clustered together, and then with the islet samples obtained from another study (4). Seven groups of genes (clusters 1-7) were identified based on tissue-specific expression patterns. “Cluster 2” comprised genes well-known as pancreatic islet-specific regulators, including *Mafa*, *Pdx1* and *Nkx6-1*. “Cluster 1”, preferentially enriched in our pancreatic islet samples (both treated and control), included hypoxia (*Hif1a*) and immune response genes (*Stat5a*, *Stat6*) and could be ascribed to the extended culture period of our samples (4 days) in comparison with those obtained from Vogel et al. (48h) (4), as supported by previous studies (25, 26). The remaining clusters were associated with brain- (cluster 3), liver/brain- (cluster 4), liver- (clusters 5 and 6) and liver/islet-enriched transcripts (cluster 7). Row-hierarchical clustering identifies groups of genes that are enriched in different tissue samples. Triplicate tissue samples were processed for Liver (L), Brain (B), pancreatic islet public datasets (P) and our INGAP-PP (I) and Control (C) pancreatic islets. (C) The gene expression pattern characteristic of each row-hierarchical clustered genes shows preferential expression in specific tissues. The boxes show the IQR of RNA levels, whiskers extend to 1.5 times the IQR or extreme values and notches indicate 95% confidence intervals of the median. (D) Barplots showing the gene expression level (TPM) for selected islet cell type-specific genes, split by biological replicates in control islet samples. Violin plots next to barplots show the single-cell expression profiles for the same genes, supporting the islet cell type-specific expression pattern (data from (7)).

**Supplementary Figure 2. Functional annotation of genes regulated by INGAP-PP treatment shows expression in non-endocrine cell types within pancreatic islets: stellate, Schwann and endothelial cells.** (A) Selected GSEA results depicting enrichment (*p*< 0.05; FDR < 0.25) of genesets associated to Schwann and endothelial cells, linked to axonal guidance and angiogenesis, respectively. NES: Normalized Enrichment Score. (B) Heatmap showing the Leading Edge Analysis of selected GSEA gene sets. Blue arrows indicate genes with expression enriched in different cell types. (C) Violin plots show the single-cell expression profiles for the genes marked with blue arrows in (B), supporting its Schwann and endothelial islet cell type-specific expression pattern (data from (7)). (D) Average gene expression fold enrichment (from RNA-seq data) for top enriched genes in selected annotations shown in (A). Bars represent means ± SEM. (E) Violin plots depicting the single-cell expression profiles for genes related with the ECM organization categories shown in **Fig. 1G**, which are consistently induced by the INGAP-PP treatment, showing preferentially enriched expression in activated pancreatic stellate cells (data from (7)). (F) UCSC Genome Browser screenshots showing illustrative examples for genomic loci associated with endocrine cell specific genes and genes potentially expressed in other minor islet cell populations, thus devoid of islet-specific transcription factor binding in human pancreatic islets. Original data taken from (12). In agreement with the non-endocrine islet cell type-specific expression profile for the *Col6a1*/*Col6a2* genes, its genomic locus lacked binding for any of 5 endocrine-specific transcription factors in human pancreatic islets, in stark contrast with the epigenomic landscape in the vicinity of genes selectively expressed in endocrine cells, such as *Pax6* (illustrated in panel E).

**Supplementary Figure 3. Integrative Genomic Viewer screenshots of the epigenomic landscape at loci of genes coding for transcription factors enriched among the top *de-novo* motifs found in Fig. 2E.** The active epigenomic landscape, with gene promoters enriched in ATAC-seq, H3K4me3 and H3K27ac signal, and gene bodies presenting enrichment of both H3K36me3 and H3K79me2, further supports that these genes are active in β-cells. The genomic regions visualized are shown in square brackets.

**Supplementary Figure 4. INGAP-PP upregulates a subset of genes whose downregulation is associated with IL1β-induced stress in β-cells.** (A, B) Expression pattern of transcripts that are downregulated by IL1β in INS-1 cells in: (A) INGAP-PP treated rat pancreatic islet replicates, and (B) INS-1 cells treated with either IL1β or control (DMSO). The boxes show the IQR of RNA levels, whiskers extend to 1.5 times the IQR or extreme values and notches indicate 95% confidence intervals of the median. **p<*0.05, ***p<*0.01, calculated with the Wilcoxon rank-sum test. (C) *Mlxipl* expression quantified from RNA-seq replicates of rat pancreatic islets treated with INGAP-PP or control. We also validated *Mlxipl* upregulation by INGAP-PP treatment in 3 additional replicate experiments. RT-qPCR data is normalized to *Actin* gene expression.

**Supplementary Figure 5. INGAP-PP induces the expression of genes previously unannotated in the rat genome.** (A) Integrative Genomic Viewer screenshot of the *Gnas* locus showing the epigenomic profile (in untreated INS-1 cells) and the RNA-seq pile up signal for islet, brain and liver samples analyzed in this study. This genomic locus presents 4 distinct alternative promoters for *Gnas*, labeled as promoters 1 to 4 according to the transcription levels in islets and INS-1 cells (clearly different as evaluated from the RNA-seq pile up signal) and the level of H3K4me3 and ATAC-seq signal enrichments at their respective promoters. This panel suggests that the *Gnas* variant transcribed from promoter 1 is the most highly expressed in rat β-cells. Of note, *Gnas-as2* (MSTRG.20759) is a previously unannotated, non-coding transcript expressed antisense from the *Gnas* variant expressed from promoter 1, having thus a complementary sequence to most of the other *Gnas* mRNA variants. The *Gnas* variants presented have been curated to show the most representative of the *de novo* annotations recovered by the HiSAT-StringTie pipeline. (B) *Gnas-as2* expression is downregulated by IL1β in INS-1 cells, and its expression is not restored by the IL1β+Cal treatment. (C) UCSC Genome Browser screenshot of the same *Gnas* locus presented in ***A***, showing the genes identified in the NCBI Rattus Norvegicus Annotation Release 106. The location of MSTRG.20759 (*Gnas-as2*), mapped by the Blat tool of the Genome Browser, is shown in dark red. (D) UCSC Genome Browser screenshots of the MSTRG.20759 locus showing the neighbor genes annotated in the NCBI Mus musculus Annotation Release 109 (mm39) and the NCBI Homo sapiens Annotation Release 110 (hg38). The location of MSTRG.20759, mapped by the Blat tool of the Genome Browser, is shown in black (in contrast to the validated genes, shown in blue). Alignments of nucleotides use color to highlight differences as follows: red indicates that the mouse/human genome and query sequence have different bases at this position; orange indicates that the query sequence has an insertion (or the mouse/human genome has a deletion / alignment gap) at this point; and purple indicates that the query sequence extends beyond the end of the alignment. (E) Integrative Genomic Viewer screenshots for the novel lncRNAs induced by INGAP-PP in rat treated pancreatic islets. Note that most of the non-annotated transcripts with active promoters in INS-1 did also show H3K36me3 and H3K79me2 enrichments along the gene bodies, consistently with actively transcribed genes. Barplots on the right of each screenshot show the expression levels for the indicated genes as quantified from RNA-seq in INS-1 cells treated with either IL1β, IL1β+Cal or control (DMSO). The genomic regions visualized in the screenshot panels are shown in square brackets. Data are expressed as mean ± SEM. * *p*<0.05, ** *p*<0.01 calculated using EdgeR.

**Supplementary Figure 6. UCSC Genome Browser screenshot of the equivalent MSTRG.16201 (*Ri-lnc1*) locus presented in Fig. 4F, showing the genes identified in the NCBI Homo sapiens Annotation Release 110 (hg38).** The location of *Ri-lnc1*, mapped by the Blat tool of the Genome Browser, is shown in grey. Alignments of nucleotides use color to highlight differences as follows: red indicates that the human genome and query sequence have different bases at this position; orange indicates that the query sequence has an insertion (or the human genome has a deletion / alignment gap) at this point; and purple indicates that the query sequence extends beyond the end of the alignment. The genomic region visualized in the screenshot is shown in square brackets.

**Supplementary Figure 7. Integrative Genomic Viewer screenshot of the MSTRG.4242 locus showing the epigenomic profile (in untreated INS-1 cells) and the RNA-seq pile up signal for islet, brain and liver samples analyzed in this study.** The putative promoter of MSTRG.4242 was not marked by H3K4me3 in INS-1 cells but presented a strong Neurod1 binding site near its TSS and clear enrichments for H3K4me1 and H3K27ac signal in INS-1 cells, suggesting that it could be a transcribed enhancer region. The barplot of the right shows that the expression of MSTRG.4242 is downregulated by IL1β in INS-1 cells, and its expression is not restored by the combined IL1β+Cal treatment. The genomic region visualized is shown in square brackets.

**Supplementary Figure 8. *Ri-lnc1* is associated with the subset of mature β-cells.** (A) Feature plots showing the expression pattern for the lncRNAs reported in this work that present a pan-endocrine profile, and for MSTRG.4242. Islet cells clustered as in **Fig. 5A**. (B) UMAP plot of 28,894 single cell transcriptomes taken from the β-cell subset in **Fig. 5A**. Colours in the UMAP on the right highlight β-cell clusters identified with default resolution setting. (C) Violin plots showing expression for wellknown mature β-cell markers *Ins1*, *Slc2a2* and *Mafa*, as well as *Ri-lnc1*, in β-cells clustered as in (B). (D) Feature plots showing varying expression of *Slc2a2*, *Mafa*, *Ri-lnc1* and *Mt1* among clusters.

**References**

1. Lacy, P.E., and Kostianovsky, M., Method for the isolation of intact islets of Langerhans from the rat pancreas. *Diabetes* 16 (1967) **16**: 35-9. doi: 10.2337/diab.16.1.35

2. Linde, S., Hansen, B., and Lernmark, å., Preparation of stable radioiodinated polypeptide hormones and proteins using polyacrylamide gel electrophoresis, Methods in Enzymology, Academic Press, 1983, pp. 309-335.

3. Pertea, M., Kim, D., Pertea, G.M., Leek, J.T., and Salzberg, S.L., Transcript-level expression analysis of RNA-seq experiments with HISAT, StringTie and Ballgown. *Nature Protocols* 11 (2016) **11**: 1650-1667. doi: 10.1038/nprot.2016.095

4. Vogel, J., Yin, J., Su, L., Wang, S.X., Zessis, R., Fowler, S., et al., A Phenotypic Screen Identifies Calcium Overload as a Key Mechanism of β-Cell Glucolipotoxicity. *Diabetes* 69 (2020) **69**: 1032. doi: 10.2337/db19-0813

5. Krzywinski, M., Schein, J., Birol, I., Connors, J., Gascoyne, R., Horsman, D., et al., Circos: an information aesthetic for comparative genomics. *Genome Res* 19 (2009) **19**: 1639-45. doi: 10.1101/gr.092759.109

6. Subramanian, A., Tamayo, P., Mootha, V.K., Mukherjee, S., Ebert, B.L., Gillette, M.A., et al., Gene set enrichment analysis: a knowledge-based approach for interpreting genome-wide expression profiles. *Proc Natl Acad Sci U S A* 102 (2005) **102**: 15545-50. doi: 10.1073/pnas.0506580102

7. Baron, M., Veres, A., Wolock, S.L., Faust, A.L., Gaujoux, R., Vetere, A., et al., A Single-Cell Transcriptomic Map of the Human and Mouse Pancreas Reveals Inter- and Intra-cell Population Structure. *Cell Syst* 3 (2016) **3**: 346-360 e4. doi: 10.1016/j.cels.2016.08.011

8. Wolf, F.A., Angerer, P., and Theis, F.J., SCANPY: large-scale single-cell gene expression data analysis. *Genome Biol* 19 (2018) **19**: 15. doi: 10.1186/s13059-017-1382-0

9. Vivoli, A., Ghislain, J., Filali-Mouhim, A., Angeles, Z.E., Castell, A.-L., Sladek, R., et al., Single-Cell RNA Sequencing Reveals a Role for Reactive Oxygen Species and Peroxiredoxins in Fatty Acid-Induced Rat β-Cell Proliferation. *Diabetes* 72 (2023) **72**: 45-58. doi: 10.2337/db22-0121

10. Langmead, B., Trapnell, C., Pop, M., and Salzberg, S.L., Ultrafast and memory-efficient alignment of short DNA sequences to the human genome. *Genome Biol* 10 (2009) **10**: R25. doi: 10.1186/gb-2009-10-3-r25

11. Cebola, I., Rodriguez-Segui, S.A., Cho, C.H., Bessa, J., Rovira, M., Luengo, M., et al., TEAD and YAP regulate the enhancer network of human embryonic pancreatic progenitors. *Nat Cell Biol* 17 (2015) **17**: 615-26. doi: 10.1038/ncb3160

12. Pasquali, L., Gaulton, K.J., Rodriguez-Segui, S.A., Mularoni, L., Miguel-Escalada, I., Akerman, I., et al., Pancreatic islet enhancer clusters enriched in type 2 diabetes risk-associated variants. *Nat Genet* 46 (2014) **46**: 136-43. doi: 10.1038/ng.2870

13. Robinson, J.T., Thorvaldsdóttir, H., Winckler, W., Guttman, M., Lander, E.S., Getz, G., et al., Integrative genomics viewer. *Nature Biotechnology* 29 (2011) **29**: 24-26. doi: 10.1038/nbt.1754

14. Zhang, Y., Liu, T., Meyer, C.A., Eeckhoute, J., Johnson, D.S., Bernstein, B.E., et al., Model-based analysis of ChIP-Seq (MACS). *Genome Biol* 9 (2008) **9**: R137. doi: 10.1186/gb-2008-9-9-r137

15. Oropeza, D., Cigliola, V., Romero, A., Chera, S., Rodríguez-Seguí, S.A., and Herrera, P.L., Stage-specific transcriptomic changes in pancreatic α-cells after massive β-cell loss. *BMC Genomics* 22 (2021) **22**: 585. doi: 10.1186/s12864-021-07812-x

16. Langmead, B., and Salzberg, S.L., Fast gapped-read alignment with Bowtie 2. *Nat Methods* 9 (2012) **9**: 357-9. doi: 10.1038/nmeth.1923

17. Quinlan, A.R., and Hall, I.M., BEDTools: a flexible suite of utilities for comparing genomic features. *Bioinformatics* 26 (2010) **26**: 841-2. doi: 10.1093/bioinformatics/btq033

18. de Hoon, M.J., Imoto, S., Nolan, J., and Miyano, S., Open source clustering software. *Bioinformatics* 20 (2004) **20**: 1453-4. doi: 10.1093/bioinformatics/bth078

19. Saldanha, A.J., Java Treeview--extensible visualization of microarray data. *Bioinformatics* 20 (2004) **20**: 3246-8. doi: 10.1093/bioinformatics/bth349

20. Heinz, S., Benner, C., Spann, N., Bertolino, E., Lin, Y.C., Laslo, P., et al., Simple Combinations of Lineage-Determining Transcription Factors Prime cis-Regulatory Elements Required for Macrophage and B Cell Identities. *Molecular Cell* 38 (2010) **38**: 576-589. doi: 10.1016/j.molcel.2010.05.004

21. Wei, Z., Yoshihara, E., He, N., Hah, N., Fan, W., Pinto, A.F.M., et al., Vitamin D Switches BAF Complexes to Protect β Cells. *Cell* 173 (2018) **173**: 1135-1149.e15. doi: https://doi.org/10.1016/j.cell.2018.04.013

22. Kent, W.J., BLAT--the BLAST-like alignment tool. *Genome Res* 12 (2002) **12**: 656-64. doi: 10.1101/gr.229202

23. Altschul, S.F., Gish, W., Miller, W., Myers, E.W., and Lipman, D.J., Basic local alignment search tool. *J Mol Biol* 215 (1990) **215**: 403-10. doi: 10.1016/S0022-2836(05)80360-2

24. Kang, Y.J., Yang, D.C., Kong, L., Hou, M., Meng, Y.Q., Wei, L., et al., CPC2: a fast and accurate coding potential calculator based on sequence intrinsic features. *Nucleic Acids Res* 45 (2017) **45**: W12-W16. doi: 10.1093/nar/gkx428

25. Negi, S., Jetha, A., Aikin, R., Hasilo, C., Sladek, R., and Paraskevas, S., Analysis of beta-cell gene expression reveals inflammatory signaling and evidence of dedifferentiation following human islet isolation and culture. *PLoS ONE* 7 (2012) **7**: e30415. doi: 10.1371/journal.pone.0030415

26. Haga, J., Sato, N., Anazawa, T., Kimura, T., Kenjo, A., Gotoh, M., et al., Comprehensive analysis of gene expression of isolated pancreatic islets during pretransplant culture. *Fukushima J Med Sci.* 67 (2021) **67**: 17-26. doi: 10.5387/fms.2020-25
