# Supplementary figures and images for: Transcriptional signature of islet neogenesis-associated protein peptide-treated rat pancreatic islets reveals induction of novel long non-coding RNAs
Source: Front Endocrinol (Lausanne). 2023 Sep 29;14:1226615. doi: 10.3389/fendo.2023.1226615 (PMC10570750; doi:10.3389/fendo.2023.1226615)

Figure S1

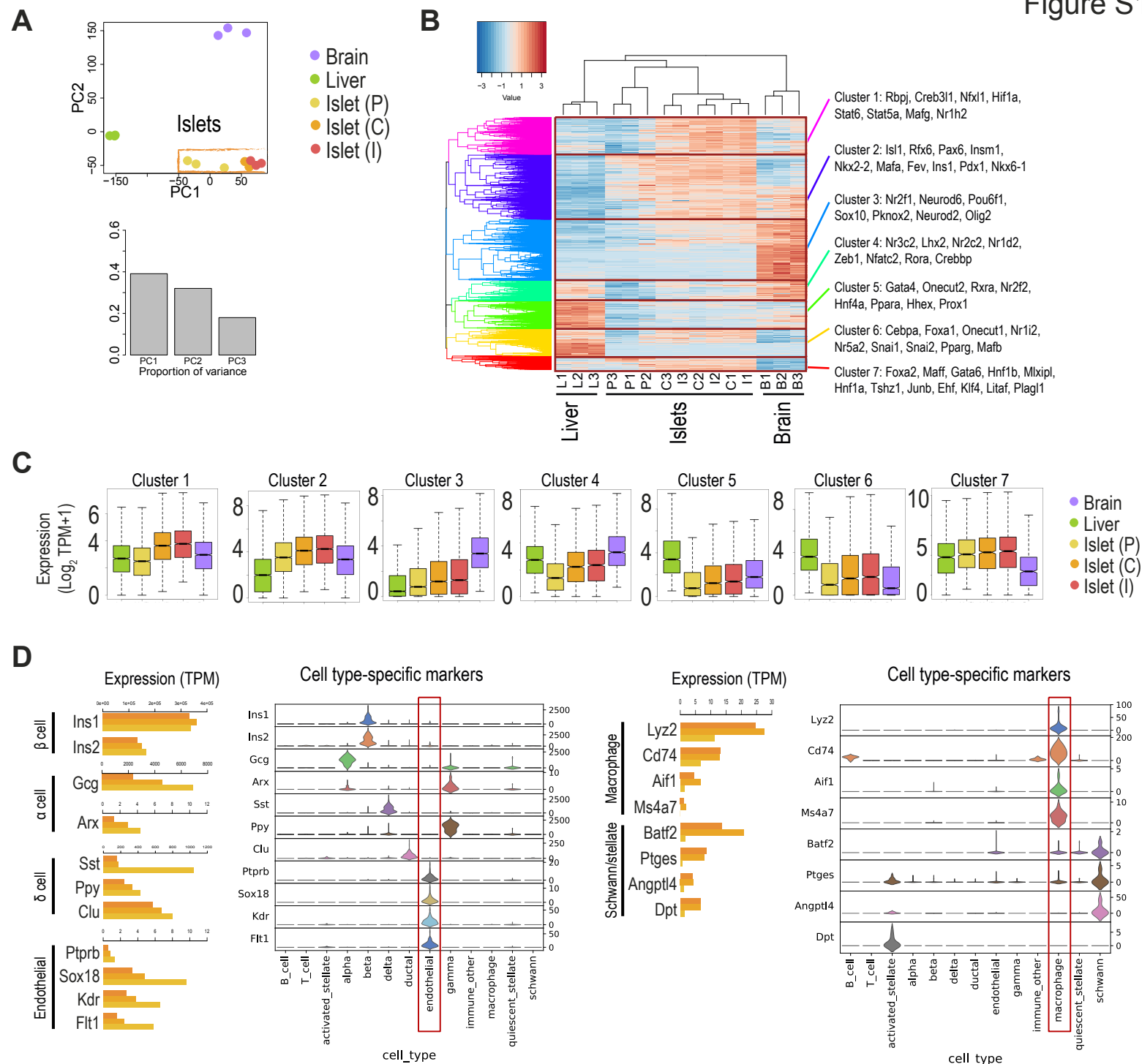

**A**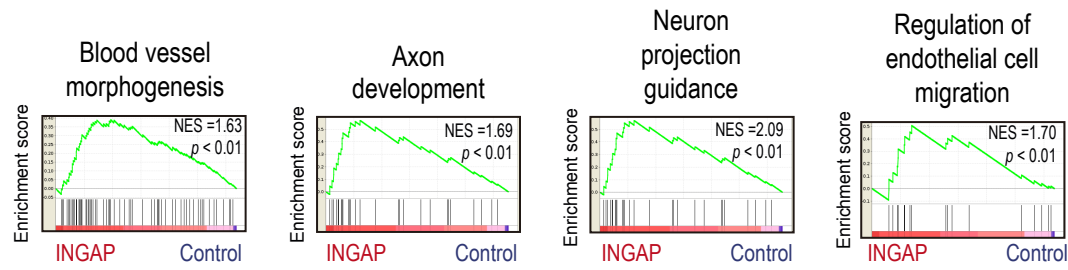**B**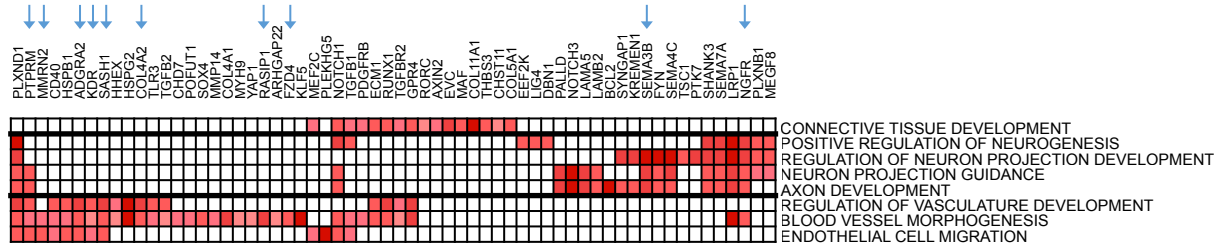**C**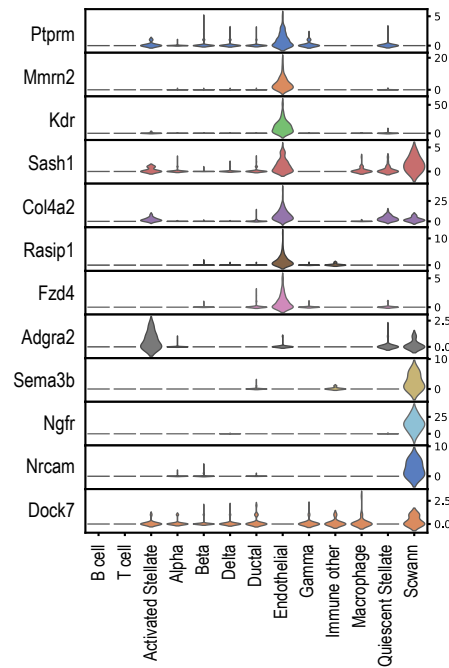**D**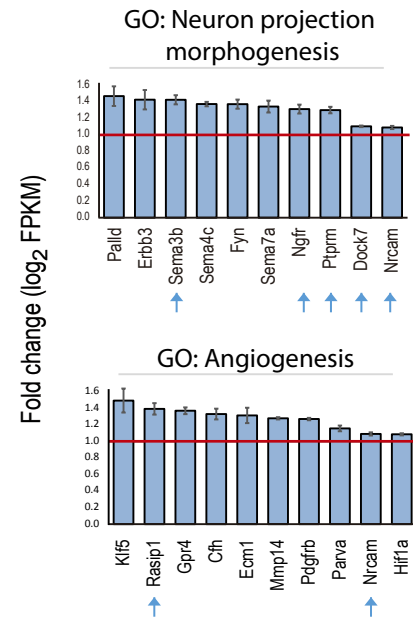**E**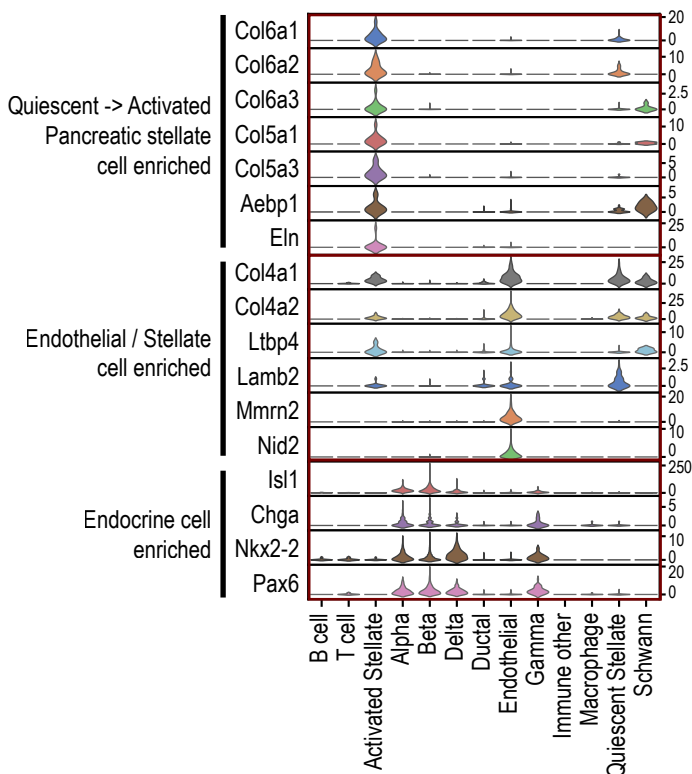**F**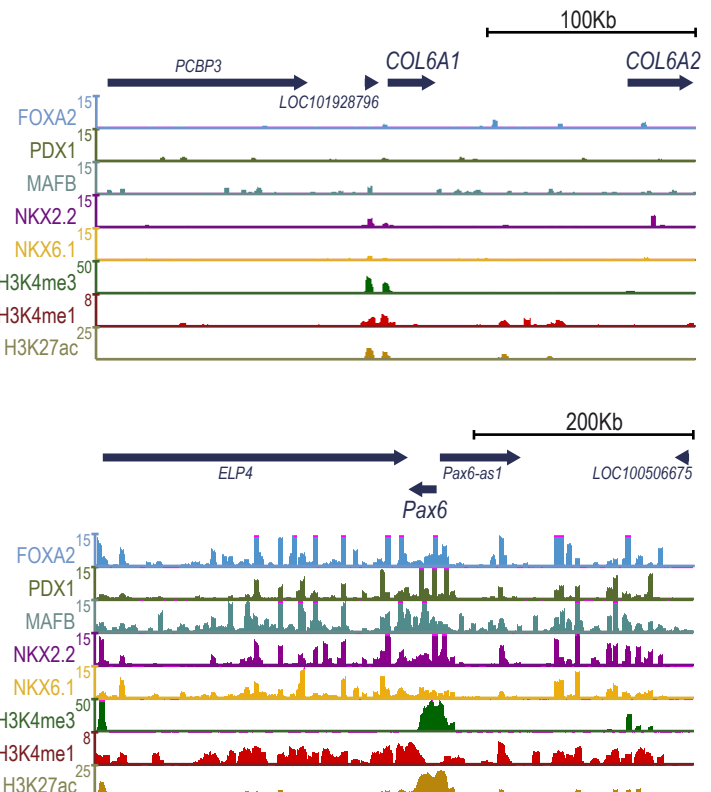

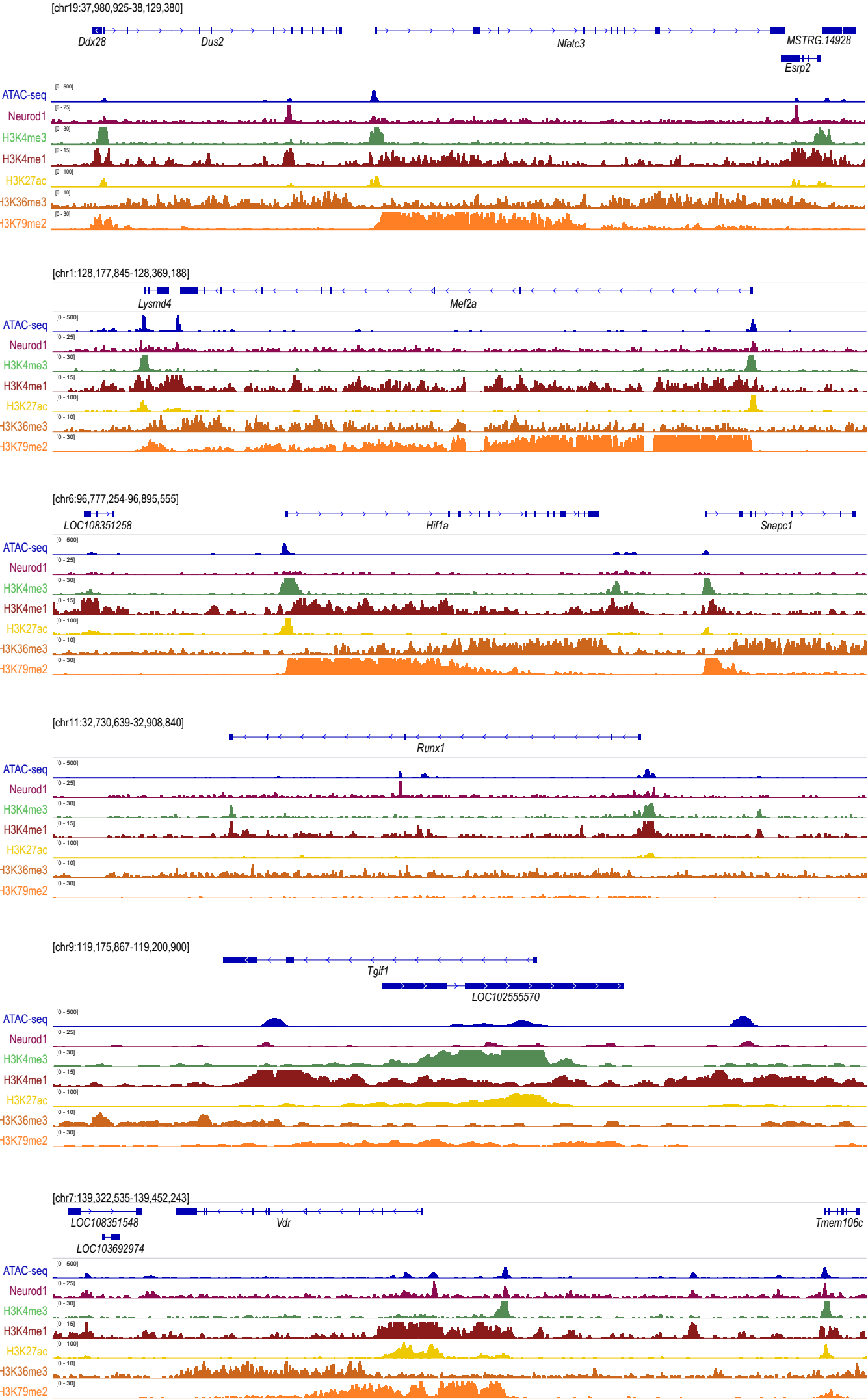

Figure S4

**A**

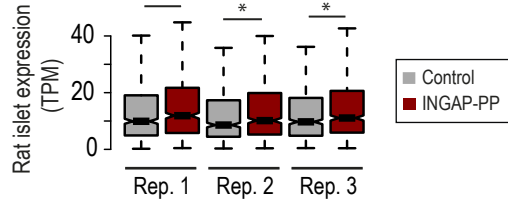

**B**

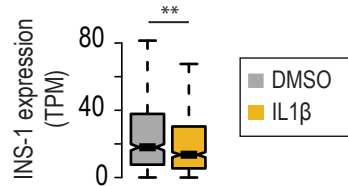

**C**

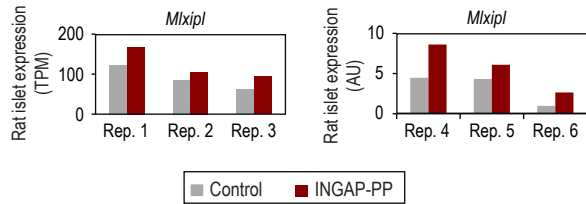

A

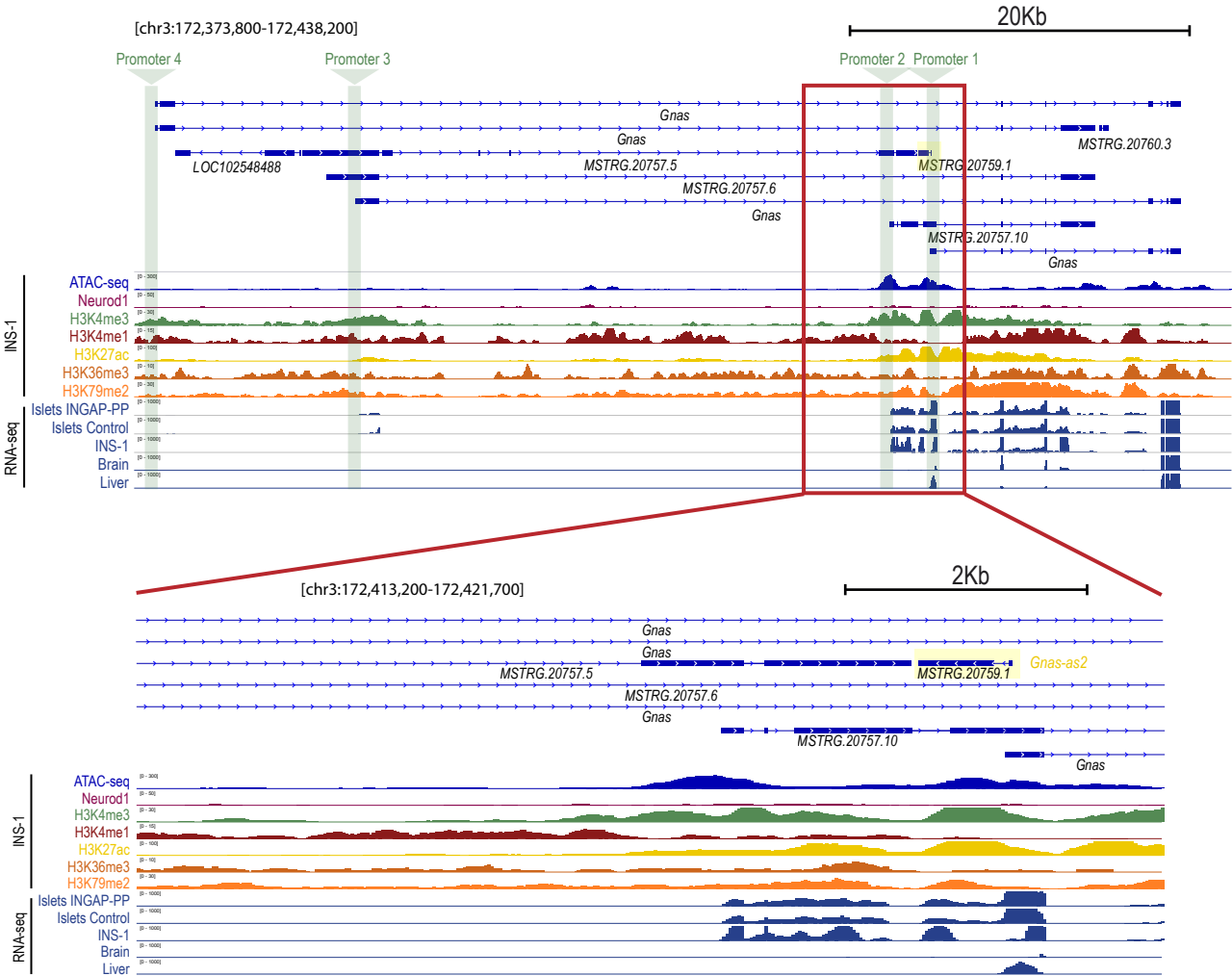

B

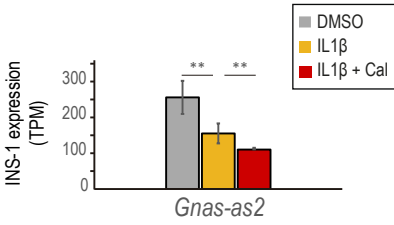

C

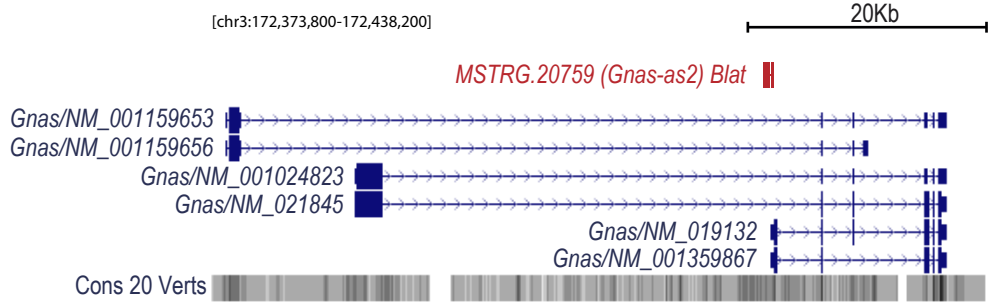

D

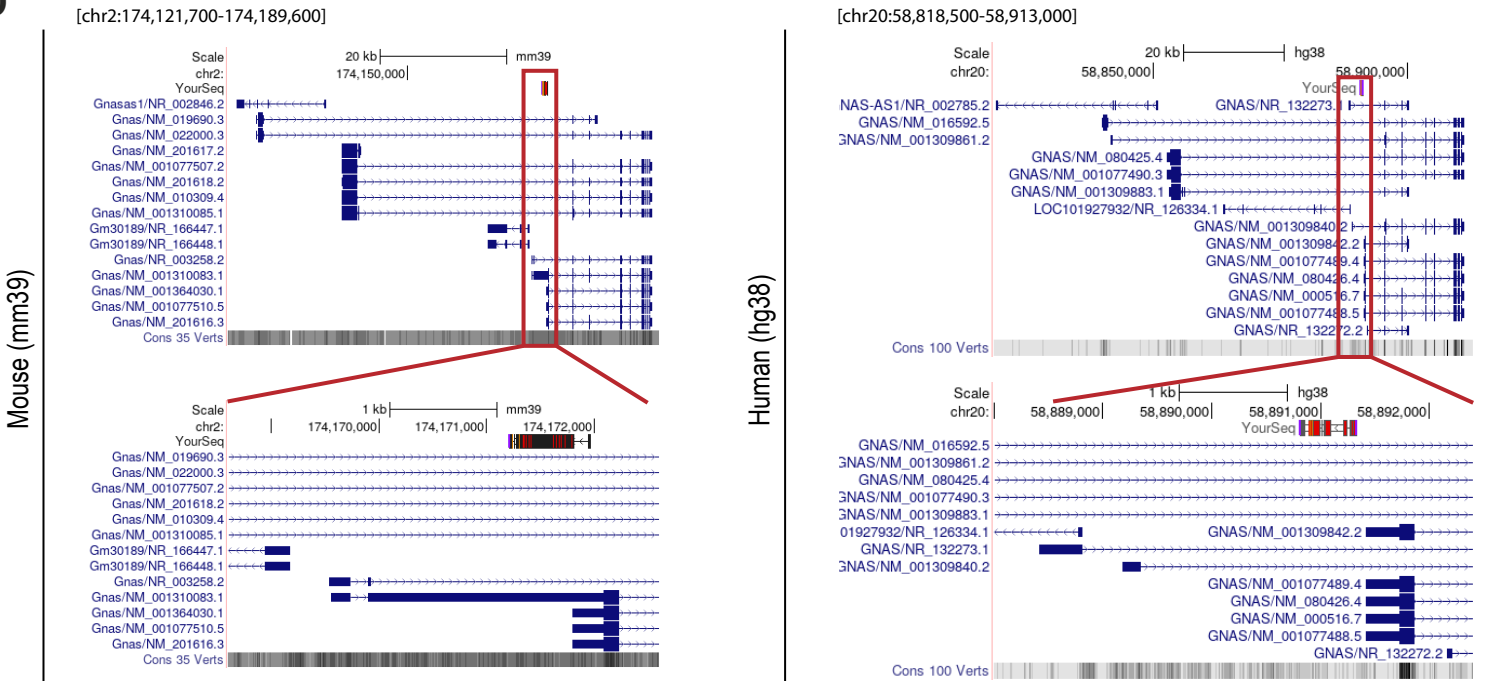

E

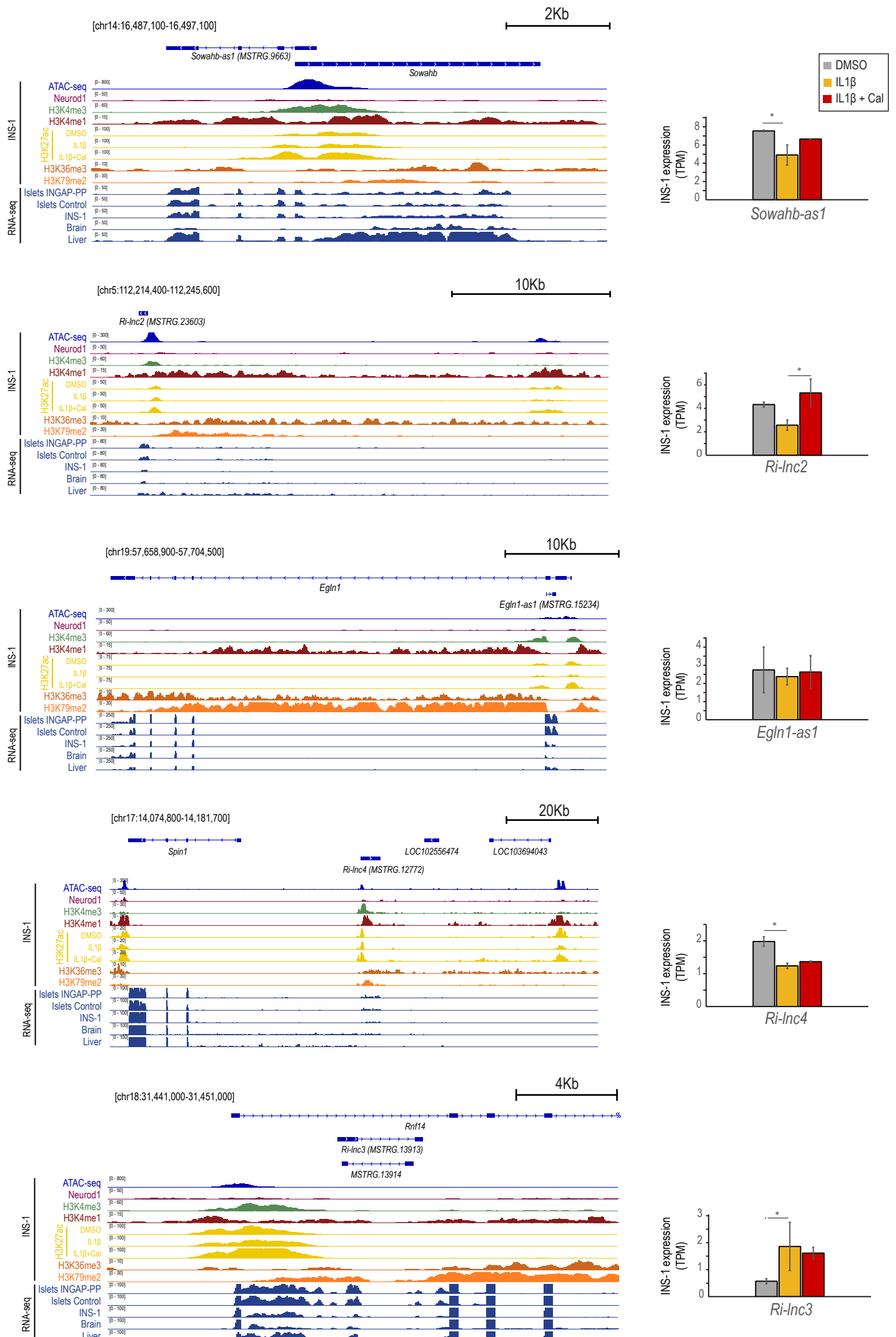

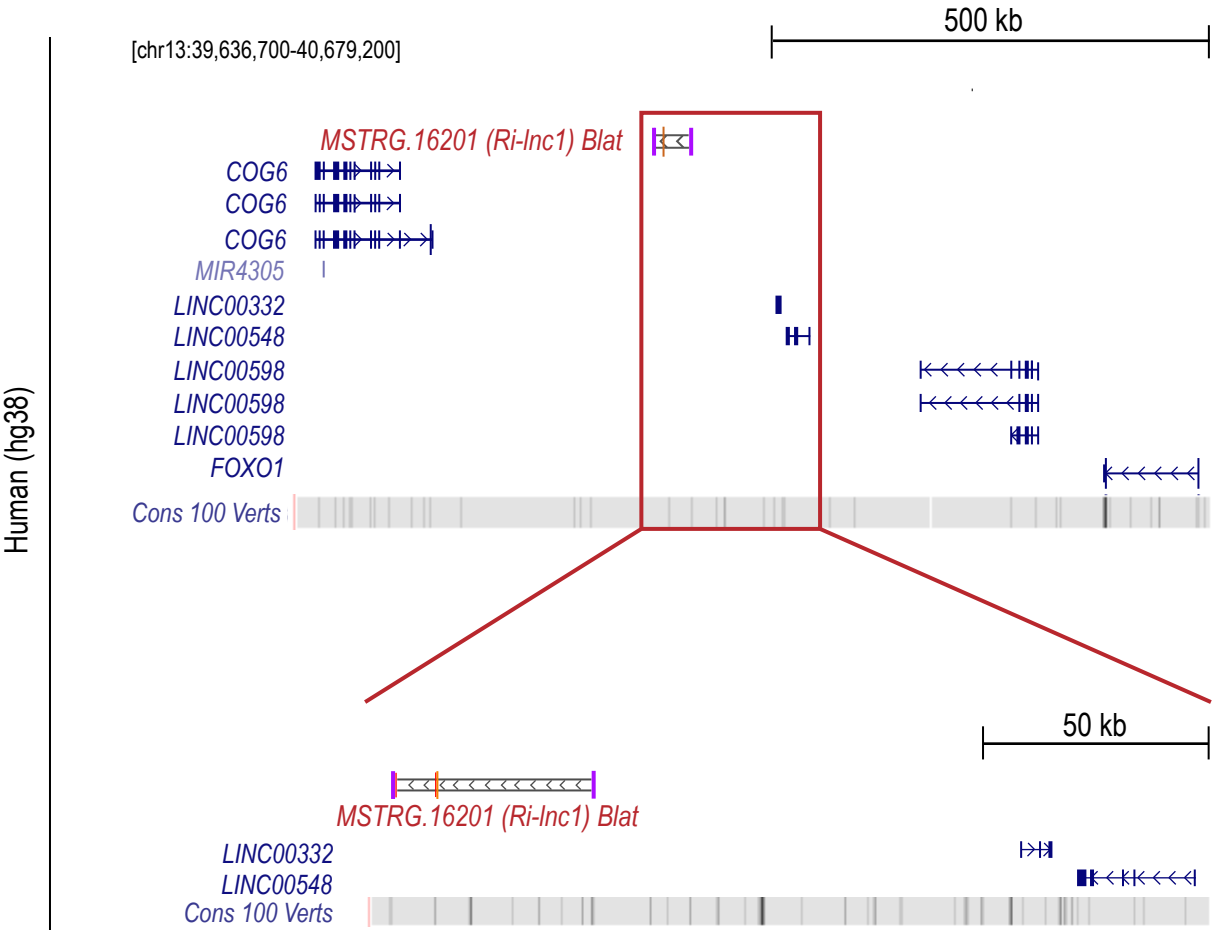

Figure S7

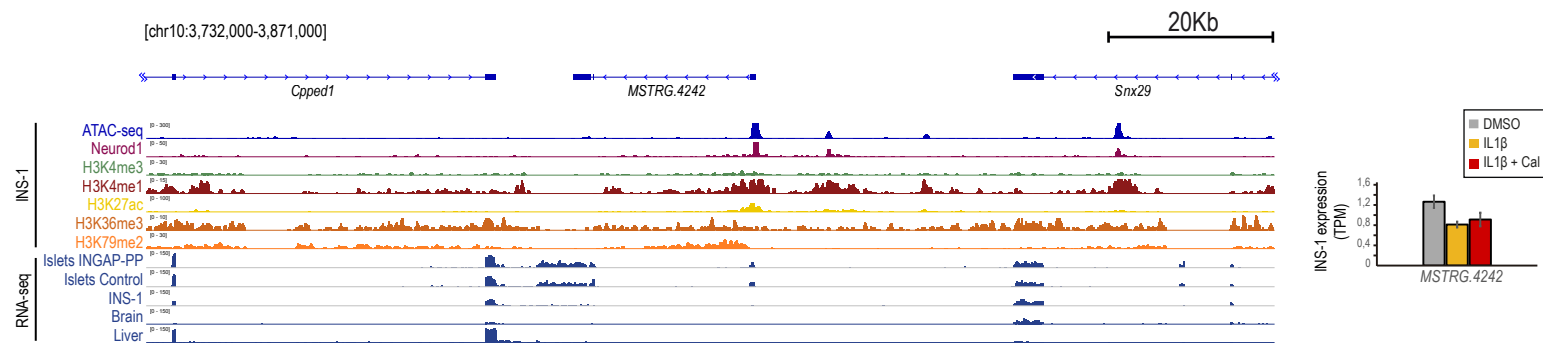

**A**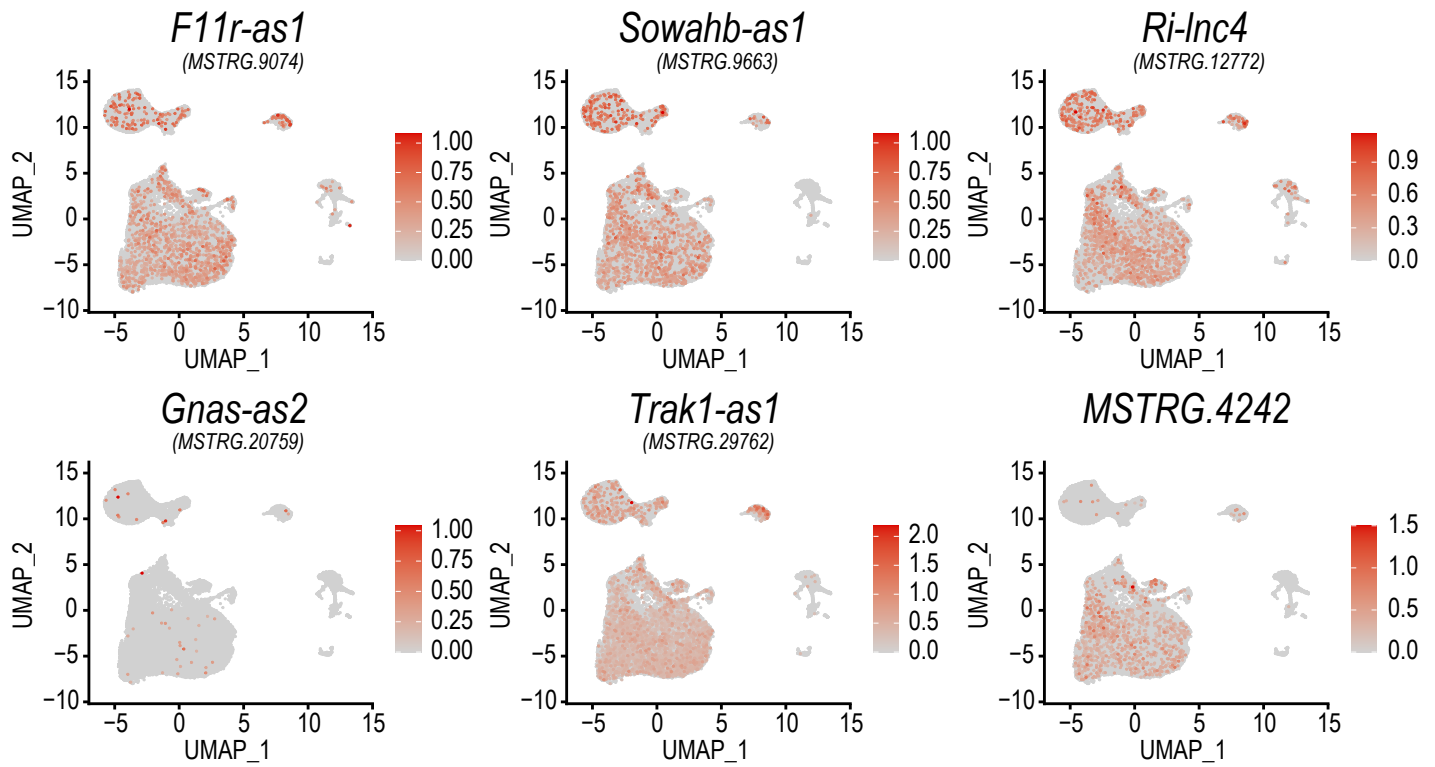**B**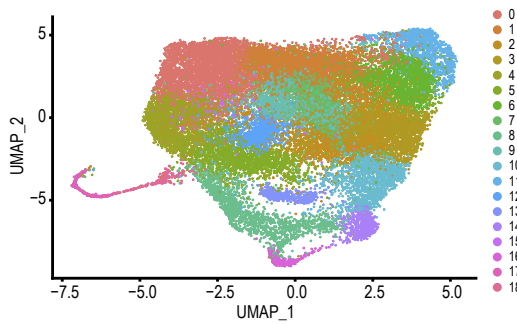**C**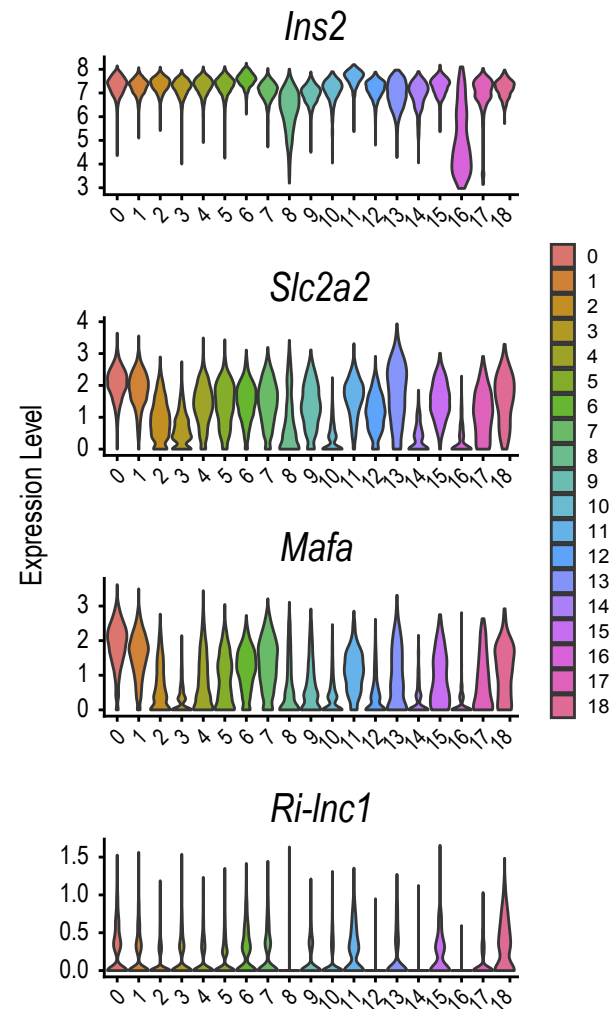**D**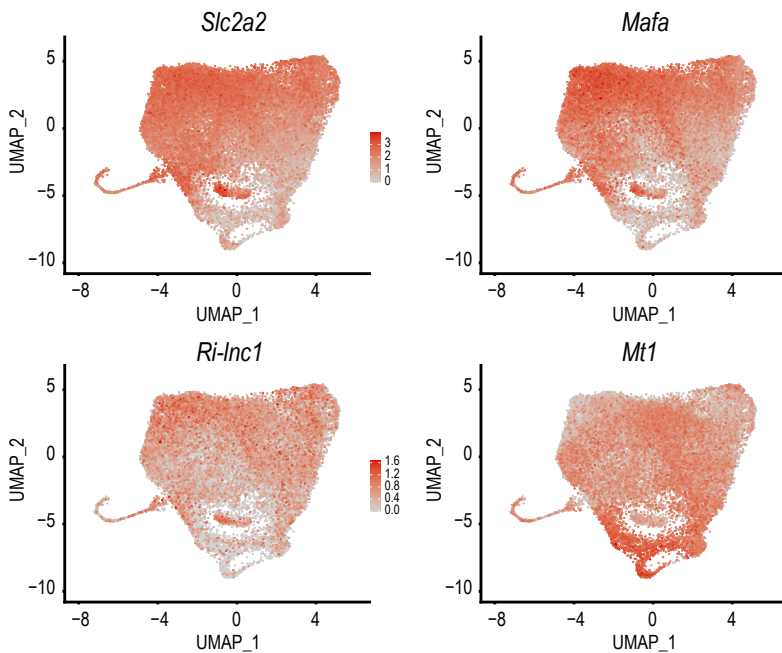

Supplement: Supplementary file 2 [file Image_1.pdf]
